# ROS production in response to high-power microwave pulses induces p53 activation and DNA damage in brain cells: Radiosensitivity and biological dosimetry evaluation

**DOI:** 10.3389/fcell.2023.1067861

**Published:** 2023-02-23

**Authors:** Juie Nahushkumar Rana, Sohail Mumtaz, Eun Ha Choi, Ihn Han

**Affiliations:** ^1^ Department of Plasma Bio Display, Kwangwoon University, Seoul, Republic of Korea; ^2^ Plasma Bioscience Research Center (PBRC), Kwangwoon University, Seoul, Republic of Korea; ^3^ Department of Electrical and Biological Physics, Kwangwoon University, Seoul, Republic of Korea

**Keywords:** high power microwaves, nanosecond pulses, reactive species, microwave p53 activation, microwave and brain, radiation safety level, hippocampal

## Abstract

**Background:** Pulsed high-power microwave (HPM) has many applications and is constantly being researched to expand its uses in the future. As the number of applications grows, the biological effects and safety level of pulsed HPM become a serious issue, requiring further research.

**Objective:** The brain is regarded as the most vulnerable organ to radiation, raising concerns about determining an acceptable level of exposure. The effect of nanosecond pulses and the mechanisms underlying HPM on the brain has not been studied. For the first time, we observed the effect of pulsed 3.5 GHz HPM on brain normal astrocytes and cancer U87 MG cells, as well as the likely mechanisms involved.

**Methods:** To generate 3.5 GHz HPM, an axial virtual cathode oscillator was constructed on pulsed power generator “*Chundoong”*. The cells were directly exposed to HPM (10, 25, 40, and 60) pulses (1 mJ/pulse), with each pulse delivered after 1 min of charging time to evaluate the dose dependent effects.

**Results:** A strong electric field (∼23 kV/cm) of HPM irradiation primarily causes the production of reactive oxygen species (ROS), altering cell viability, mitochondrial activity, and cell death rates in U87 and astrocytes at certain dosages. The ROS generation in response to HPM exposure was primarily responsible for DNA damage and p53 activation. The hazardous dosage of 60 pulses is acknowledged as having damaging effects on brain normal cells. Interestingly, the particular 25 pulses exhibited therapeutic effects on U87 cells *via* p53, Bax, and Caspase-3 activation.

**Conclusion:** HPM pulses induced apoptosis-related events such as ROS burst and increased oxidative DNA damage at higher dosages in normal cells and specific 25 pulses in cancer U87. These findings are useful to understand the physiological mechanisms driving HPM-induced cell death, as well as the safety threshold range for HPM exposure on normal cells and therapeutic effects on cancer U87. As HPM technology advances, we believe this study is timely and will benefit humanity and future research.

## 1 Introduction

The numerous applications of microwave radiation (MWR) in modern society have made them an indispensable part of our daily lives (Benford, 2008; Al_Dulamey, 2021). Because numerous forms of radiation occur in our daily living surroundings, the consequences of biological system exposure constitute an important topic of research, which encompasses both the biological impacts and the safety levels of these types of radiation. MWR-based disinfection technologies have gained popularity in recent decades due to improvements in equipment uniformity, a decrease in undesirable disinfection byproducts, and residential applications (Gubler and Hiller, 1984; Jang et al., 2021). MWR can penetrate materials, air, and vacuum by nature. These are classified as non-ionizing radiation and are found in the electromagnetic spectrum right next to infrared (Osepchuk, 1984; Polikarpov, 1998). The electromagnetic wave’s electric field is primarily responsible for heating. Beyond its home use, several other MWR applications in other disciplines have been identified (Balmori, 2009; Shaw et al., 2021). These applications heavily depend on the electromagnetic energy and frequency of the MWR (Mumtaz et al., 2020a). A few of the primary industries where MWR has had a big impact include: industrial, military, communication, radar, linear collider, accelerator, fusion heating, astronomy, and medicine (Bakhshabadi et al., 2017; Ramos et al., 2017). MWR can be utilized for medicinal applications (Tabuse, 1998; Vrba, 2005; Grenier et al., 2013). MWR sensing and imaging have been employed for tumor diagnosis (Chandra et al., 2015), early diagnoses (Bond et al., 2003), blood clot/stroke detection (Fhager et al., 2018), heart imaging (Semenov et al., 2003), bone imaging and radiofrequency source localization (Meaney et al., 2012). Continuous MWR exposure may be useful for biological activities such as wound healing (both septic and aseptic) and cerebral hematoma diagnosis (Chandra et al., 2015). Long-term exposure to MWR would cause a multitude of negative effects on the entire body, tissue, cell, and gene levels. Since electronic technology has advanced so quickly, concern over the potential health risks brought on by MWR has risen.

Disorders of energy metabolism do contribute significantly to the process of brain damage brought on by microwave radiation. The public’s interest has been sparked by MWR’s effects on brain metabolic pathways. In recent times, MWR therapy has also been used in clinics to treat various cancers (Nikawa, 2013). MWR has a variety of biological impacts on malignancies in addition to coagulation-based tumor death. Numerous studies indicate that MWR causes tumor cells to undergo apoptosis (Mumtaz et al., 2022a). A better prognosis was shown in those who received the MWR doses prior to surgery. W-band millimeter-wave radiation at a non-thermal power density (0.2 mW/cm^2^) is used to study the morphological alterations in the human lung cancer cell (H1299) (Komoshvili et al., 2020). According to the results of reported study, exposure to millimeter waves with a frequency range of 75–110 GHz alters cell shape in a dose-dependent manner. The MWR method is applicable to a wide range of applications, including lung disease diagnosis and mapping. It is mostly unknown how MWR affects brain cancer cells and normal cells. It is reasonable to suppose that using pulsed HPM-based technologies like radar, which have the potential to cause wider environmental electromagnetic pollution, will expose people to the pulses when testing, identifying, and mapping tumors. Applications for pulsed radiations, like HPM, are rapidly expanding for future life.

The human body has a weak but stable electromagnetic (EM) field, and at a certain level, this EM field could interfere with the bioelectrical processes and cause instability. The nervous system is particularly vulnerable to external EMF since it depends on bioelectricity to function (Beason and Semm, 2002; Kumar et al., 2011). It is thought that the nervous system is a significant and sensitive target for MWR. It is now well documented that MWR also has significant health-damaging consequences, notably on the nervous system (Hao et al., 2015). One of the most vulnerable target organs for microwave radiation is the brain, where mitochondrial damage manifests more quickly and severely than in other organs (Lin, 2021), with the hippocampus being particularly sensitive. The structure and operation of the nervous system can be harmed by prolonged exposure to high levels of RF energy. Long-term MWR exposure or MWR in the 860–2,450 MHz frequency range has substantial consequences on the brain, including hippocampal destruction, neurotransmitter disruption, and cognitive impairment (Wang et al., 2015; Xiong et al., 2015; Odaci et al., 2016; Terzi et al., 2016). By inducing functional and morphological lesions on natural killer (NK-92) cells, pulsed and dose-dependent MWR influence autoimmunity and immunomodulation. Apoptosis and cytotoxicity of natural killer cells induced by MWR through ERK1/2 Signaling (Zhao et al., 2017). Prolonged exposure to a 900-MHz electromagnetic field for 1 h per day has been shown to alter normal morphological cell structures, as well as metabolic parameters and an increase in apoptosis (Kerimoglu et al., 2016). Pulsed MWR at 2.856 GHz was studied for its impact on bone marrow cells, however, no significant changes in cell survival, apoptosis, or cell division were observed (Wang et al., 2015a). Overall, given the growing use of MWR in biology and other elements of daily life, it is critical to study and assess the impacts of MWR on biological systems. High-power microwaves (HPM) on the other hand, have been employed as weapons that can be used in future space and military applications. Pulsed-HPM is extremely useful to disable the electronic systems of cars that pose a threat. The increased usage of pulsed-HPM in recent decades has prompted worries about its potential biological effects, which are still being researched and many facts are unknown to science. To the best of our knowledge, and based on a review of the available literature on this topic, the effect, and mechanism of nanosecond pulses of 3.5 GHz HPM radiation on brain cancer and normal cells is mostly unknown.

In this study, we sought to determine the effect and mechanism of nanosecond pulses of 3.5 GHz HPM radiation on brain normal (astrocytes) and cancer (U-87 MG) cells. Pulsed HPM affected both healthy and brain cancer fibroblast cells. The virtual cathode oscillator (vircator) was used to generate the HPM with the frequency of 3.5 GHz and cells were directly exposed to HPM radiation with 10, 25, 40, and 60 pulses. At the cellular level, the impact of HPM is evaluated by examining metabolic viability and cell death by molecular apoptotic markers. We anticipate that this research may help develop certain factors to optimize HPM treatment as well as the frequency range at which normal human brain cells may survive.

## 2 Materials and methods

### 2.1 Pulsed HPM generator and experimental details

**FIGURE. 1:**
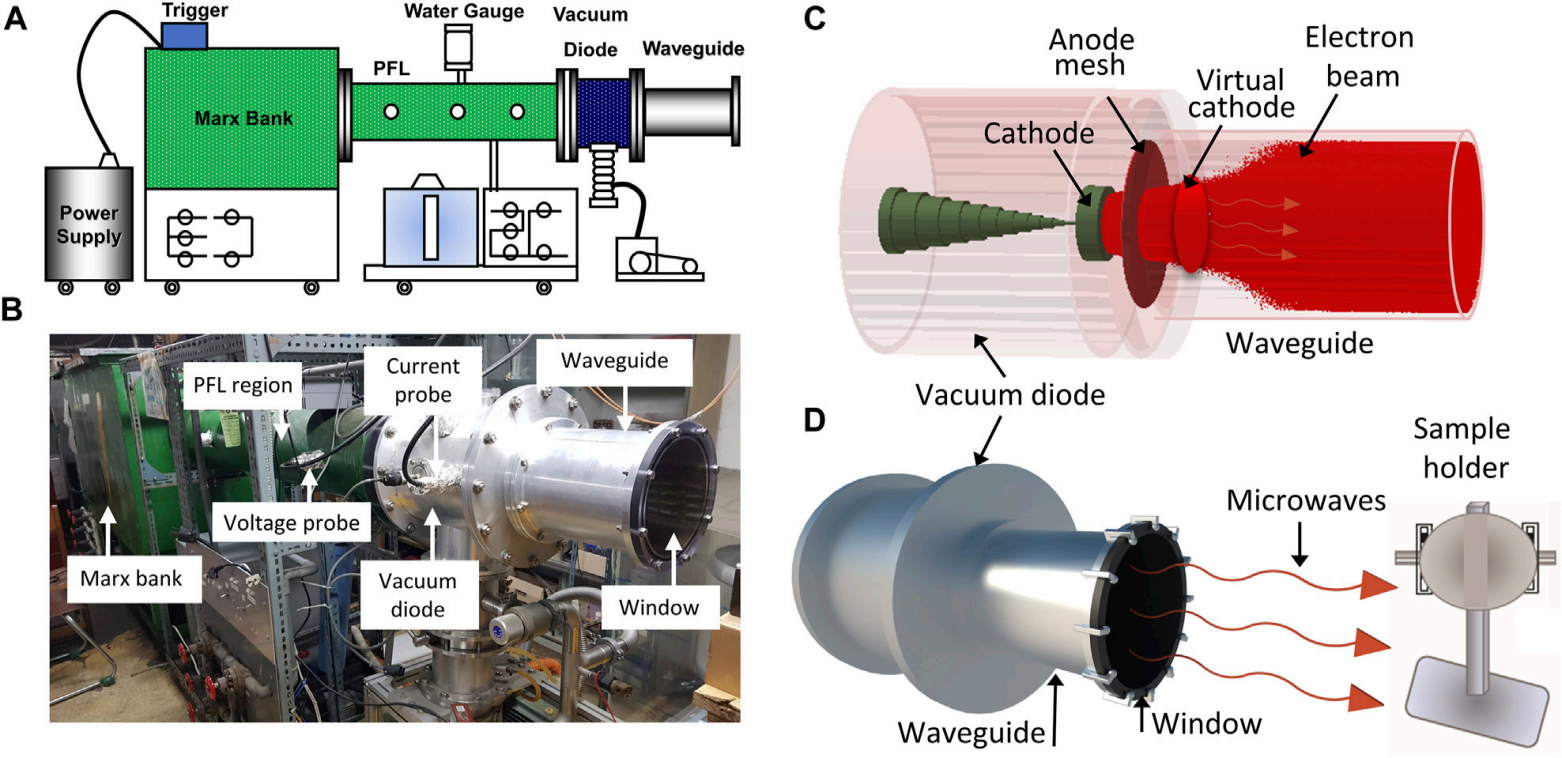
The details of the HPM device and experimental arrangements. The **(A)** and **(B)** is the schematical configurations and photograph of the pulsed high power microwave generator, *Chundoong*, and **(C)** shows the device’s internal view in simulation with beam emission and formation of VC, and **(D)** is the experimental setup, where, the samples were situated on the sample holder, 15 cm away from the output window where they were exposed with HPM pulses.

The virtual cathode oscillator (vircator), which can function at high frequencies and generate HPM with high power at a low voltage, is regarded as the greatest HPM source since it is simple to construct and comprehend. For the production of HPM, an axial vircator was built and used in this study. For this, a pulsed HPM-generator known as the *chundoong* was employed for the production of HPM which produces high power at levels ranging from several hundred megawatts to gigawatts using an intense relativistic electron beam (Mumtaz et al., 2018). The device was schematically and photographically presented in Figures 1A, B. A high-voltage pulse is produced in this device using a Marx generator, which connects 12 capacitors in series. A 20 kV DC source was used to charge each capacitor for 1 min. Each capacitor had a capacitance of up to 0.2 µF. When discharged in a series with a single trigger shot after charging, they generate a pulse with a high voltage. When the characteristic impedance of the pulse-forming line, i.e., 6.8 Ω, matched the diode chamber, which was evacuated with vacuum on $1\times 10^{-5}$ torr, the characteristics of the produced electron beam achieved the optimum conditions (Mumtaz et al., 2019). The vacuum diode region of the device was comprised of three basic components: A cathode, an anodic mesh foil, and a virtual cathode (VC). The cathode is composed of metal and has a 4.5 cm radius covered with velvet.

The anodic mesh had a radius of 10 cm, which corresponded to the inner radius of the waveguide. The guiding tube was 25 cm long and its end part was sealed with acrylic material to maintain the vacuum pressure. This window also allowed the produced HPM to propagate outside of the guiding tube, preventing surface breakdown. In our earlier work, the mechanisms by which Chundoong produces HPM were extensively described (Mumtaz et al., 2022b; Mumtaz and Choi, 2022). B-dot probe was used to measure current in the vacuum diode region, and C-dot probe was used to measure voltage in the pulse forming line region. All waveforms were sent to the electromagnetic shielding room to be observed at an oscilloscope after the microwave real signal was captured by the receiving antenna at the location of the samples. With the Fast Fourier Transform (FFT) of microwave signal, the frequency was obtained. With the envelope signal of HPM, the power of microwave was calculated (Mumtaz et al., 2019). The HPM frequency was also determined in simulation by taking the FFT of electric field, which is estimated inside the diode region with simulation time.

For treatment, the cells are grown in T75 flasks and seeded at a density of $1\times 10^{4}$ cells/ml in 96 well plates. Two groups are used in each experiment: a control group and a treatment group. The cells that were not treated aided as the control group. On the other hand, the treated groups were exposed to 10, 25, 40, and 60 pulses of HPM when the sample was placed at the sample holder, 15 cm from the output window of the HPM-generator as shown in Figures 1C, D. A thermal imager (FLUKE Ti9) is used to measure the temperature of the cell media. The temperature was measured by putting the cell media in the treatment room where the room temperature is 25°C. The temperature of the cell medium was recorded at 10, 25, 40, and 60 min after putting the cell media in two conditions: without HPM exposure (control) and with HPM exposure (treated). Similarly, the pH of the cell medium was also measured by using a pH meter (Oakton, PCTS tester TM50) in control and indicated HPM pulses. The control and treated groups were incubated for 24 h. Cells were extracted after 24 h to evaluate cell viability, ATP levels, and other biological effects of HPM on Astrocytes and U87 cell lines.

### 2.2 Estimation of reactive species in cell medium after HPM exposure

To measure the levels of reactive oxygen species (ROS) and reactive nitrogen species (RNS) in the medium, cell culture media (DMEM) were exposed with HPM using the chosen pulses (10, 25, 40, and 60). First, the ROS and RNS measurements made immediately following exposure are shown as 0 h. NO_x_ and H_2_O_2_ measurements were taken at 0 h with and without NAC. After that, the media was kept in the incubator under conditions similar to those used for cell incubation, and ROS and RNS were measured at each of the chosen times (12, 24, 48, and 72 h) after irradiation. The Quantichrom Nitric Oxide Assay Kit (D2NO-100) was used to analyze RNS (NO_x_) levels according to the instructions, while its Quantichrom Peroxide Assay Kit (DIOX—250) was utilized to detect ROS (H_2_O_2_) using the provided protocol.

### 2.3 Cell culture

The Korean Cell Line Bank sold human Brain cancer (U87) and brain normal fibroblast Astrocyte cell lines (Seoul, Korea). These cells were grown in DMEM (Cat# LM001-05; Welgene, Korea) media with 10% fetal bovine serum, 100 g/mL streptomycin, and 100 U/mL penicillin in a humidified incubator with 5% CO_2_ at 37°C. Cells were subculture in a medium every two to 3 days. It is generally recognized that MWR has a wide range of applications in both brain imaging and tumor monitoring. Science is still unsure of how HPM affects brain cells. The goal of using these cell lines was to examine the biological effects of HPM on brain cells.

### 2.4 Metabolic viability

Using the Alamar blue dye, the metabolic viability of U87 and Astrocytes cells was assessed (DAL1025; Thermo Fisher Scientific, Waltham, MA, United States). Keeping the cell density at $1\times 10^{4}$ cells per 100 μl, the cells were planted in 96-well plates. With control and treated groups, experiments were carried out in at least three copies. The fluorescence emission of the Alamar blue dye was measured using a BioTek plate reader with an excitation and emission wavelengths of 540 nm and 600 nm, respectively.

### 2.5 Intercellular ATP measurement

Cellular ATP as a measure of cellular health was measured using the Cell Titer-Glo Assay following the manufacturer’s instructions [Promega (cat no. G7572)]. For this, $1\times 10^{4}$ cells per well (100 µl) were seeded in 96-well plates. Post 24 h of incubation, cells were supplemented with an equal volume of prewarmed reagent and incubated for 1 h at 37°C. After incubation, luminescence was measured using a microplate reader.

### 2.6 Extracellular ATP measurement

Extracellular ATP called Damage-associated molecular patterns (DAMPs) was measured using the Cell Titer-Glo Assay following the manufacturer’s instructions [Promega (cat no. G7572)]. For this, $1\times 10^{4}$ cells per well (100 µl) were seeded in 96-well plates. Post 24 h of incubation, supernatant of cells transfer into another 96 well plate, and cells were supplemented with an equal volume of prewarmed reagent and incubated for 1 h at 37°C. After incubation, luminescence was measured using a microplate reader.

### 2.7 Flow cytometric analysis for cell death

To determine the levels of cell death in cells treated with HPM-irradiated solutions, cells were seeded at a density of $2\times 10^{5}$ cells in 6 well plate. The cells were treated and incubated for 24 h. The single cell suspensions thus obtained were assessed and analyzed by flow cytometry. The pellet was resuspended in PI and Annexin staining solution provided in the kit (FITC Annexin V Apoptosis Detection Kit: BD 556547) and incubated for 15 min in ice. Then cells are subjected to analysis in a flow cytometer.

### 2.8 Intracellular ROS detection

The U87 MG and Astrocytes cells were seeded by keeping their density of $5\times 10^{4}$ per round glass coverslip in 12-well plates, and they were then incubated for 24 h at 37°C with 5% CO_2_. Cells were then subjected to HPM at various treatment pulse lengths (control, 25, and 60 pulses). To measure intracellular ROS, 2′,7′Dichlorodihydrofluorescein diacetate (H2DCFDA, Invitrogen, CA, United States) was obtained following the manufacturer’s instructions. By using laser scanning confocal microscopy (Zeiss, LSM 510, Little Rock, AR, United States) at a magnification of ×40, fluorescence pictures were obtained. According to the manufacturer’s instructions, the experimental procedures were carried out. NAC (5 mM) was used as a ROS scavenger.

### 2.9 Molecular analysis (PCR)

Quantitative real time PCR is an advanced reaction that can be performed with RNA extraction. The molecular analysis was carried out using cDNA as a template. Caspase-3, Caspase-8, PARP, MCP-1, and Fas apoptosis markers were examined for an underlying mechanism of in-death cross talk after HPM exposure. The primers tested (Bionix, Seoul, Korea) after 24 h of incubation in astrocytes, most 60 pulses and U 87 -MG 25 pulses were of primary interest for deep mechanism as well as next step performance. Table 1 contains the primer sequences provided in Supplementary Information.

**TABLE 1:** The list of used primers. GAPDH was kept as an endogenous control for the qPCR experiment.

Primer name	Forward sequence	Reverse sequence
ART	GGG​ATG​CCA​CTG​CTT​GTT​ATG​AC	CTG​TCC​ACT​CGG​ACC​TGT​TAG​C
ATM	GCA​GAT​GAC​CAA​GAA​TGC​AA	GGC​CTG​CTG​TAT​GAG​CAA​AT
Chk1	CTTTGGCTTGGCAACAGT	CCAGTCAGAATACTCCTG
Chk2	CTC​GGG​AGT​CGG​ATG​TTG​AG	GAG​TTT​GGC​ATC​GTG​CTG​GT
MCP1	CTG​CTC​ATA​GCA​GCC​ACC​TT	CAG​ATC​TCC​TTG​GCC​ACA​AT
Caspase-8	AAG​CAA​ACC​TCG​GGG​ATA​CT	GGG​GCT​TGA​TCT​CAA​AAT​GA
PARP	GCT​CCC​AGG​AGT​CAA​GAG​TG	TCA​GGT​CGT​TCT​GAG​CCT​TT
Caspase-3	CAT​ACT​CCA​CAG​CAC​CTG​GTT​A	ACT​CAA​ATT​CTG​TTG​CCA​CCT​T
p53	GCC​CCT​CCC​AGC​ATC​TTA​TC	AAA​GCT​GTT​CCG​TCC​CAG​TAG
Fas	ATA​AGC​CCT​GTC​CTC​CAG​GT	TGG​AAG​AAA​AAT​GGG​CTT​TG
GAPDH	ATG​GGG​AAG​GTG​AAG​GTC​G	GGG​GTC​ATT​GAT​GGC​AAC​AAT​A

### 2.10 Protein analysis

Cells were seeded in 6 well plate containing cell densities at $2\times 10^{5}$ cells/ml in each well after plasma treatment at a selective period cell were incubated at 24 h. After incubation cells were subjected to harvest for Protein samples. After protein extraction, its quantification was done. Protein was analyzed by Western blotting for the expression level of proteins with the corresponding specific antibodies. In this experiment, various antibodies are used to confirm DNA damaged by p53, and Caspase 3 with control GAPDH was used.

### 2.11 Statistical evaluation

The mean standard error of three independent experiments was plotted using the Microsoft Excel software (Microsoft office 365) and Graph Pad Prism. The Student’s t-test was used to determine significance. When p was less than 0.05, the differences were considered statistically significant (*p* values: *p 0.05, **p 0.01, ***p 0.001).

## 3 Results

### 3.1 Properties of 3.5 GHz pulsed HPM

In the vircator source, HPM was produced from a VC formed inside the guiding tube (Mumtaz et al., 2020b; Mumtaz et al., 2021) after the emission of an intense relativistic electron beam (REB). The formation of VC and phase space REB was monitored in simulation and findings were provided in Supplementary Figure S1. The samples (astrocytes and U-87 MG cells) were positioned in a sample holder which is 15 cm away from the window (Figure 1D). The cells were exposed to HPM radiation using 10, 25, 40, and 60 pulses of electromagnetic energy “*E*,” which is estimated as E=P×t/2≅1mJ/pulse , where *P* is the amount of HPM approaching the sample and t≅60 ns is the pulse duration. As a result, the sample received 1 mJ of EM energy every minute at each trigger pulse.

**FIGURE. 2:**
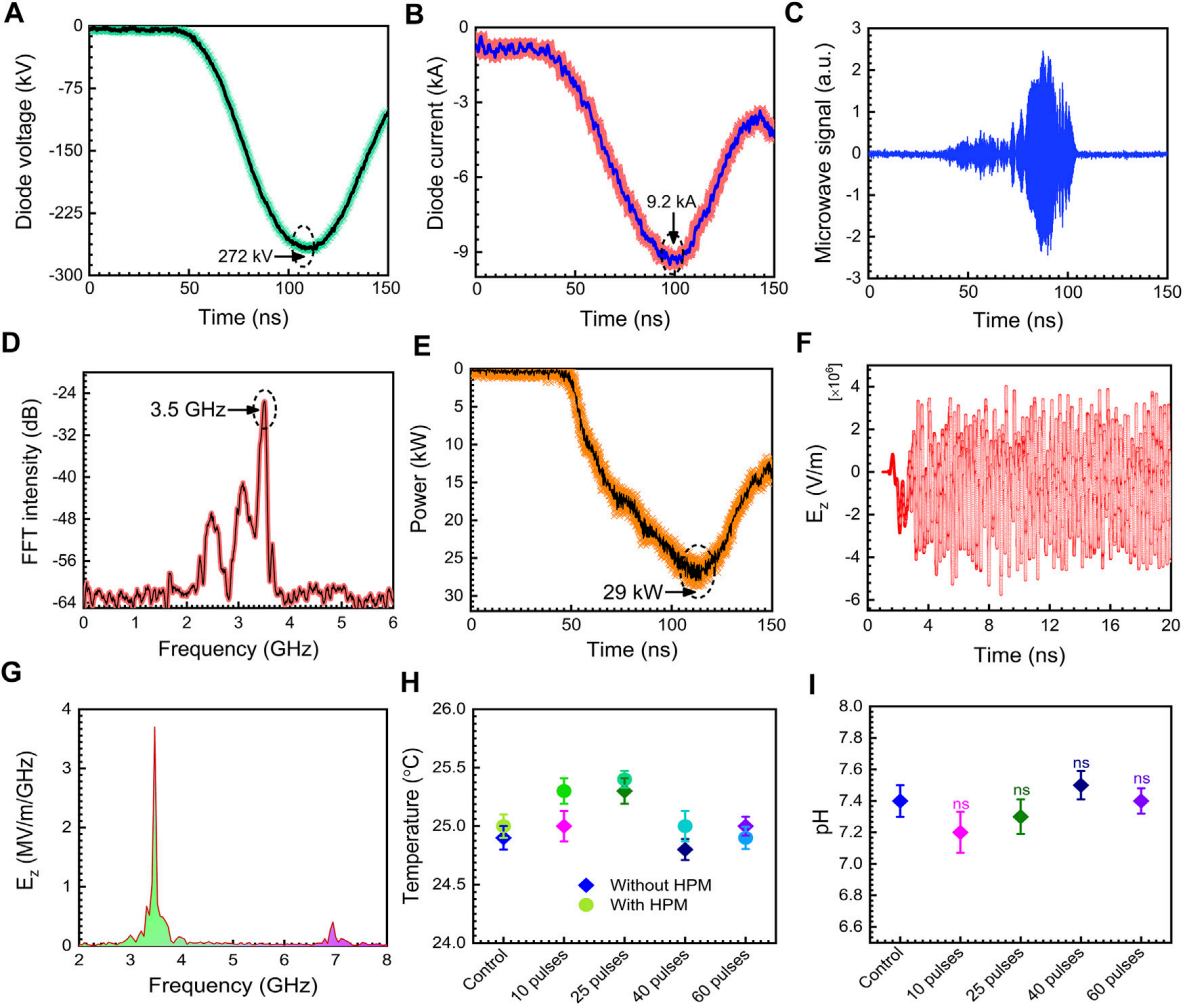
The physical properties of HPM. **(A)** diode voltage, **(B)** diode current, **(C)** microwave real signal, **(D)** measured microwave frequency, **(E)** measured microwave envelope signal, **(F)** axial electric field inside waveguide region from PIC simulation, **(G)** the calculated frequency from the FFT of the axial electric field in PIC simulation, **(H)** temperature of the media without and with microwave treatment, **(I)** pH of the cell medium before and after HPM irradiation.

The physical parameters of the 3.5 GHz pulsed HPM were tested and examined, and the results are displayed in Figure 2. The diode voltage as shown in Figure 2A rises to its maximum value of 272 kV. Figure 2B depicts the diode current with a peak value of 9.2 kA. Figure 2C represents the real HPM signal measured during the experiment. Figure 2D illustrates the dominating frequency of the produced HPM, which is determined by the FFT of the microwave real signal depicted in Figure 2C. In this work, 3.5 GHz was determined to be the dominating frequency. Figure 2E shows the HPM envelope signal which is measured at the position of the sample during the experiment. The sample was treated to HPM at a power of 29 kW on average, which equates to 1 mJ EM energy delivered to cells at each pulse. In *Chundoong*, the HPM power of 29 kW remains nearly constant as the axial distance from the window (z = 0) to the sample location (z = 15 cm) (Mumtaz et al., 2018).

The electric field strength, E_max_, from the HPM energy flowing Poynting vector has been determined to be ∼23 kV/cm in this case. In this experiment, the Poynting power density is calculated to be 12 kW/cm^2^. At each HPM pulse, an electric field with a strength of ∼23 kV/cm interacts with the media and cells. Figure 2F depicts the axial electric field E_z_ from the particle in cell (PIC) simulation code, “MAGIC” inside the drift tube area. Inside the guiding tube, the highest magnitude of E_z_ is determined to be 75 kV/cm. The 1/r dependency of field pattern and power loss owing to HPM spreading would reduce the magnitude of the electric field to 23 kV/cm at the sample position (z = 15 cm) from 70 kV/cm in the vacuum drift tube area (r is the distance between the acrylic exit window of the HPM and the target sample). Figure 2G shows the frequency spectrum which is calculated by the FFT of the axial electric field Ez given in Figure 2H. According to the simulation, the major frequency is 3.5 GHz, which is in good agreement with the measured frequency in the experiment (Figure 2D). The mode of HPM is a critical aspect in understanding its nature. We detected the emission mode of HPM in PIC simulation and results were provided in Supplementary Figure S2. Based on the data obtained, it is assumed that the TM_01_ is the dominating emission mode in this work which is identical to previously reported simulation results (Choi et al., 2000; Mumtaz et al., 2018). The temperature of the medium was measured without and with HPM exposure, and the findings demonstrate no change in temperature in the selected doses, as shown in Figure 2H. The pH of the cell medium was measured after HPM irradiation. Figure 2I depicts the measured pH in control and after being subjected to 10, 25, 40, and 60 pulses of HPM. The pH of the cell media remained constant following HPM exposure.

**FIGURE 3:**
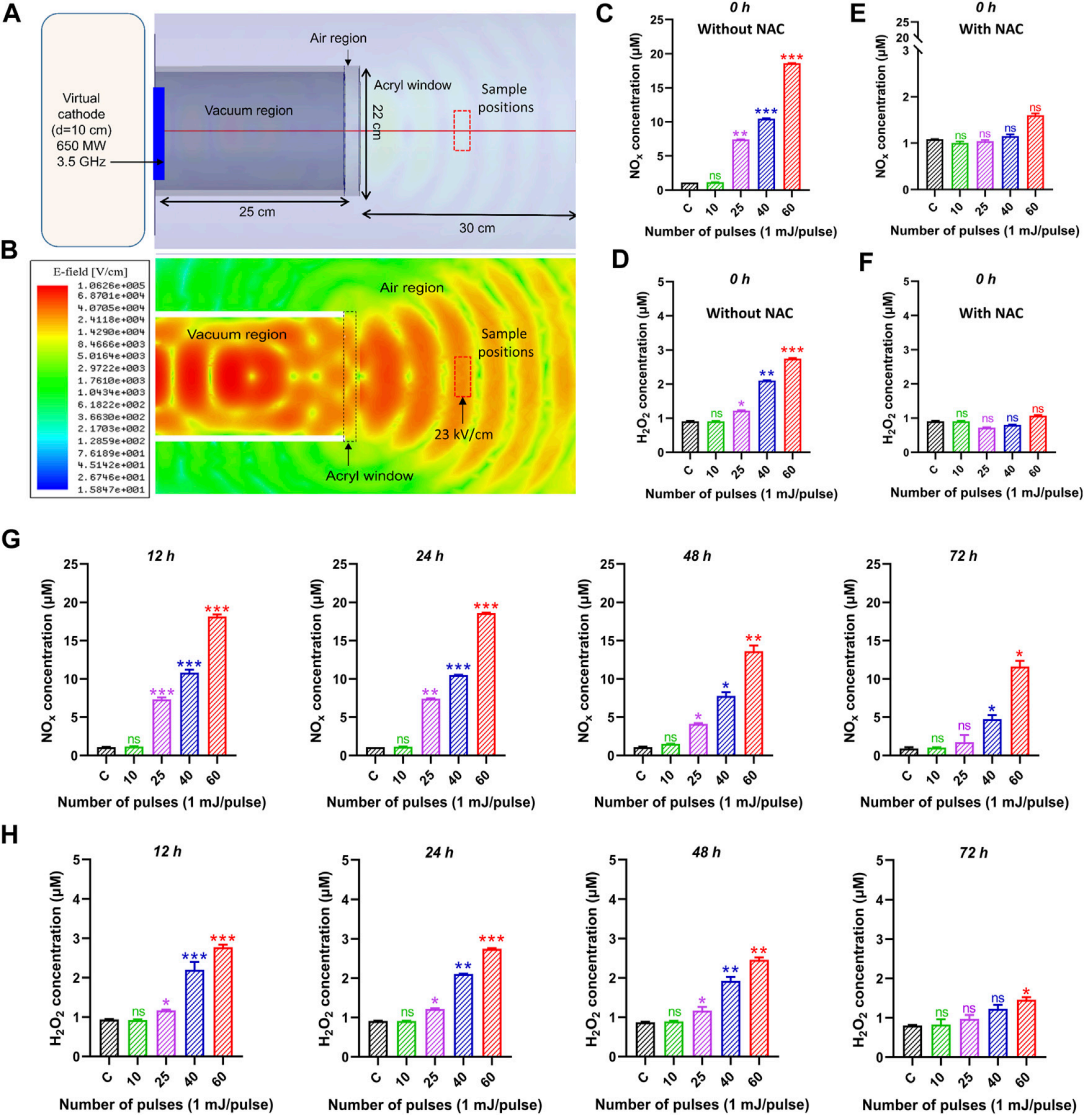
The electric field profile and reactive species inside the medium after HPM treatment. **(A)** Schematic of the device during HFSS simulation, **(B)** obtained electric field profile from HFSS simulation, **(C, D)** is NOx and H_2_O_2_ concentration in control and irradiated cell medium at indicated doses 0 h after exposure. **(E, F)** indicates the NOx and H_2_O_2_ with NAC scavenger at 0 h. The **(G, H)** indicates the NO_x_ and H_2_O_2_ concentrations at all selected incubation periods 12, 24, 48, and 72 h, respectively. Significance between control and treated groups are shown by *p < 0.05, **p < 0.01, and ***p < 0.001.

The electric field distributions at z = 15 cm from the output window was obtained using the HFSS (High-frequency structure simulator) code, as shown in Figures 3A, B. The electric field at the sample position (atmospheric region) was obtained as ∼23 kV/cm. When this electric field interacted with the cell medium, nitrogen oxygen species (NOx) and hydrogen peroxide (H_2_O_2_) were produced which might be responsible for various potential biological effects and upregulation of apoptotic gene expression levels at a specific amount of ROS and H_2_O_2_. Figure 3C demonstrates the NO_x_ concentration measured inside the medium. The NO_x_ concentration was measured immediately after the exposure of the pulsed HPM and indicated as 0 h after treatment. The obtained results indicate that the NO_x_ concentration was non-significant after 10 pulses. When the media was irradiated to 25, 40, and 60 pulses, the NO_x_ content rose dramatically to 8.5, 11, and 18 μM, respectively; Figure 3D displays the H_2_O_2_ concentration inside the medium. The H_2_O_2_ concentration remained non-significant after 10 pulses. However, when the medium was exposed to 25, 40, and 60 pulses, the H_2_O_2_ content slightly increased to 1.2, 2.3, and 2.7 µM, respectively. Additionally, we measured the ROS concentration at 0 h after adding a well-known ROS scavenger (NAC), and the results show that the ROS concentration dropped to non-significant levels as shown in Figures 3E, F. Moreover, The H_2_O_2_ and NO_x_ levels were measured in all selected incubation times (12, 24, 48, and 72 h) after exposure and results were provided in Figures 3G, H. Prior to keeping the exposed media, the NO_x_ and H_2_O_2_ levels that were measured at the time of exposure (0 h) were first recorded. After that, the ROS and RNS levels were calculated at 24, 48, and 72 h following irradiation. The levels of NO_x_ and H_2_O_2_ were found to be lower at 48 and 72 h after treatment, but for the first 24 h after exposure, they were nearly equivalent.

### 3.2 Preliminary effects of HPM on the cytotoxicity of human brain normal and brain cancer cells

**FIGURE 4:**
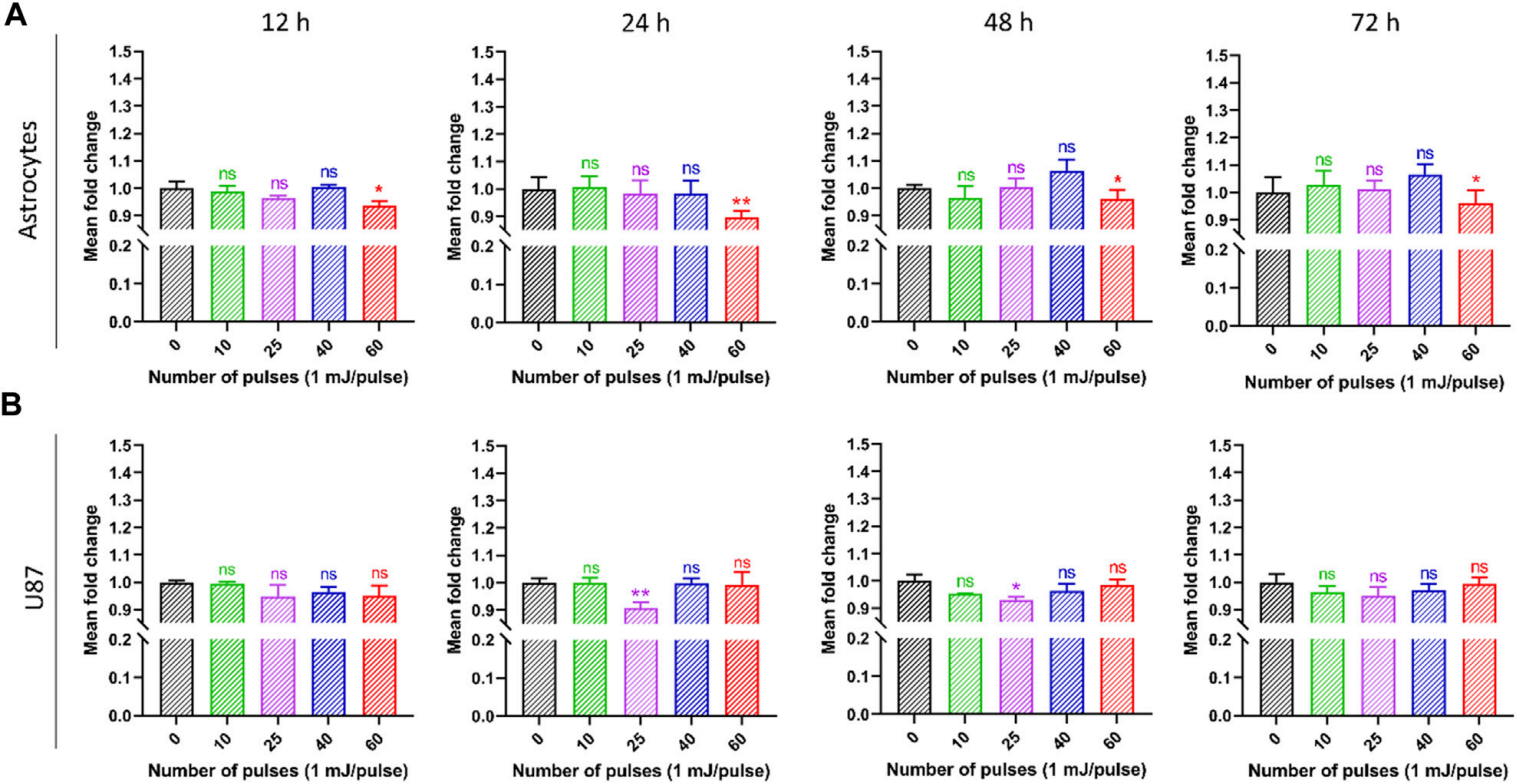
The effects of pulsed HPM on the viability of normal and malignant cells in the brain. The pulsed HPM was applied to normal (astrocyte) and malignant (U87) brain cells (10, 25, 40, and 60 pulses; 01 mj/pulse). The cell viability was assessed 12, 24, 48, and 72 h following exposure to the indicated HPM pulses, and the findings were compared to the control. The astrocyte and U87 viabilities are shown in **(A, B)**, respectively, from 12 to 72 h (*n* = 3). Microsoft Excel was used to compute the significance (MS Office 365). Differences between treatment groups are shown by *p < 0.05, **p < 0.01, and ***p < 0.001.

Human brain normal (astrocyte) and brain cancer (U87) cells were exposed to HPM radiations in this study, and their cytotoxicity was assessed using the Alamar blue assay. Both cell lines were exposed from low to high dosages of HPM pulses (1 mJ/pulse) to investigate the effect, and results were observed at 12, 24, 48, and 72 h post-exposure. Figure 4A depicts the viability of astrocyte cell lines in control and treated groups, as well as specified incubation times. The viability of the astrocyte stays unaltered after 12 h of incubation up to 40 pulses of HPM radiation, whereas 60 pulses demonstrate a modest decline in viability. A similar pattern was found after 24, 48, and 72 h, with a substantial reduction in astrocyte viability at 60 pulses on a 24-h incubation time. On the other hand, brain cancer U87 was found to be unchanged at 12 h incubation time as shown in Figure 4B. Interestingly, 25 pulses of HPM in U87 were shown to dramatically reduce cell viability 24 h after irradiation. The impact appears to be diminished after 48 h and becomes non-significant after 72 h of incubation. The 25 pulses were shown to be efficient in decreasing U87 viabilities for up to 24 and 48 h. The high dosages of HPM, 60 pulses, act as a stimulant for the U87. HPM has previously been shown to have proliferative effects on skin cancer at high doses for up to 24 h of incubation (Mumtaz et al., 2020a).

### 3.3 Effect of 3.5 GHz-HPM on the mitochondrial ATP levels

**FIGURE 5:**
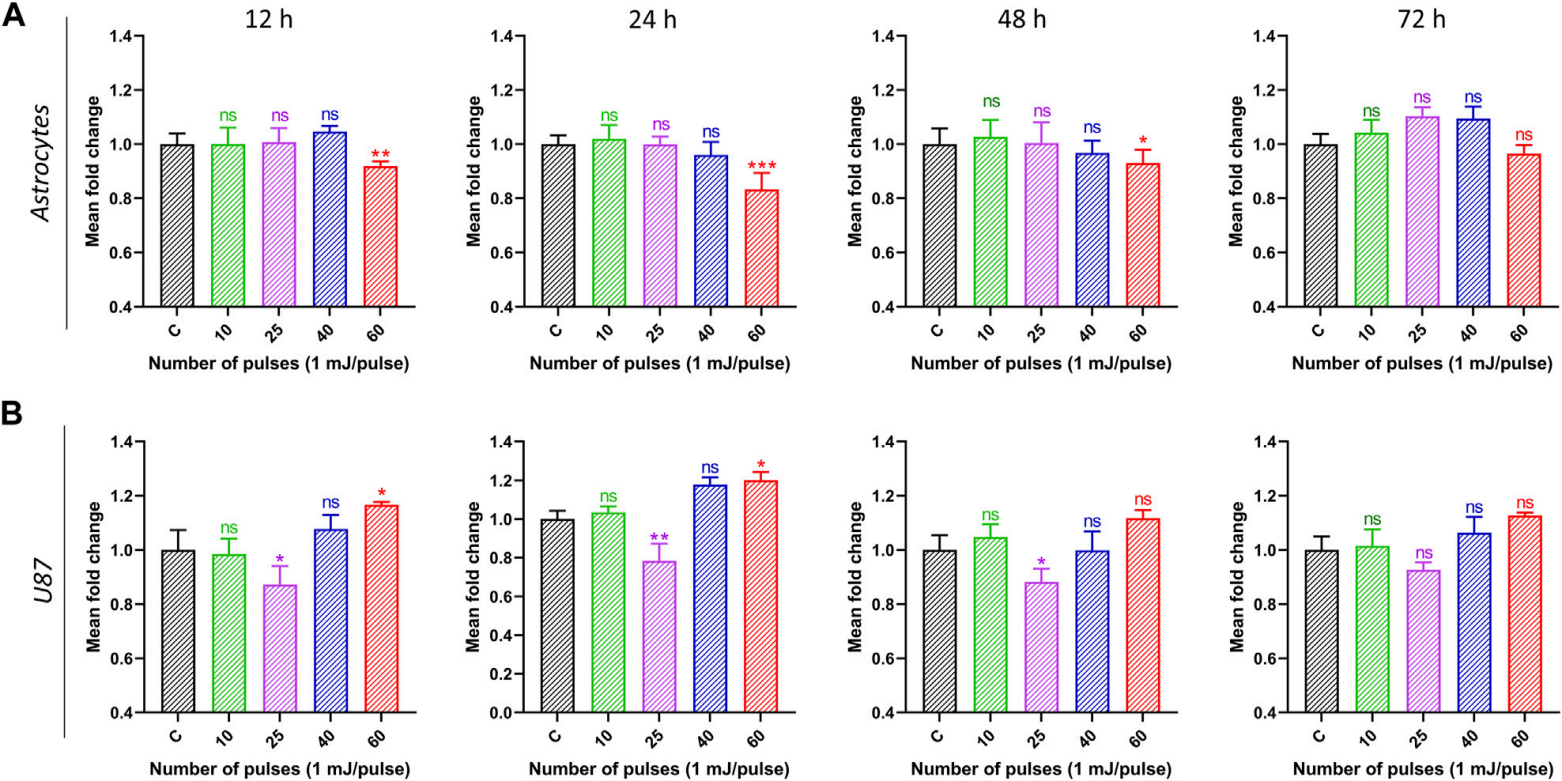
The impact of pulsed HPM on the levels of intercellular ATP in normal and cancerous brain cells. Normal (astrocyte) and malignant (U87) brain cells were treated with pulsed HPM (10, 25, 40, and 60 pulses; 01 mj/pulse). ATP levels were measured at 12, 24, 48, and 72 h after exposure to the specified HPM pulses and compared to the control. The astrocyte and U87 Intercellular ATP levels from 12 to 72 h (*n* = 3) are presented in **(A, B)**, respectively. The significance was calculated using Microsoft Excel (MS Office 365). *p < 0.05, **p < 0.01, and ***p < 0.001 indicate differences between treatment groups.

Figure 5 shows the intercellular ATP levels after HPM exposure. Both cell lines were subjected to low to high doses of HPM pulses (1 mJ/pulse) to evaluate intercellular ATP levels, and results were obtained at 12, 24, 48, and 72 h after exposure. Figure 5A depicts the ATP of astrocyte cell lines in control and treated groups, as well as specified incubation times. After 12 h of incubation, the ATP levels of the astrocyte remain unchanged until 40 pulses of HPM radiation, whereas 60 pulses show a considerable reduction in ATP levels. A similar trend was seen after 24, 48, and 72 h, with a significant drop in astrocyte ATP levels at 60 pulses on a 24-h incubation. Interestingly, 25 pulses of HPM in brain cancer U87 were observed to significantly lower ATP levels 24 h post-exposure as shown in Figure 5B. After 48 h, the impact appears to be decreased and becomes non-significant after 72 h of incubation. The 25 pulses were shown to be effective in lowering U87 ATP levels after 24 h.

### 3.4 Extracellular ATP levels

**FIGURE 6:**
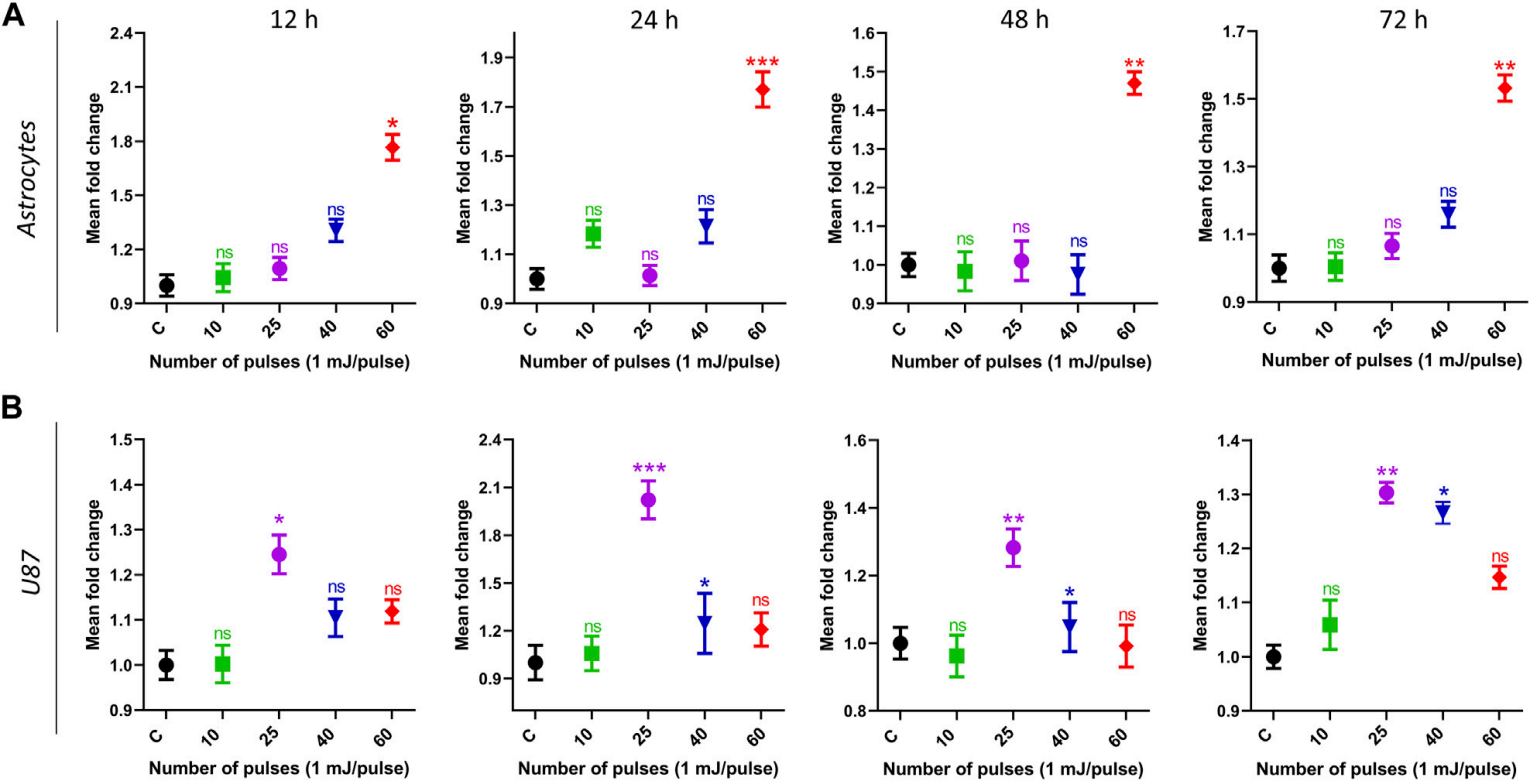
The extracellular ATP levels were measured in media. Normal (astrocyte) and malignant (U87) brain cells were treated with pulsed HPM (10, 25, 40, and 60 pulses; 01 mj/pulse). ATP levels were measured at 12, 24, 48, and 72 h after exposure to the specified HPM pulses and compared to the control. The astrocyte and U87 extracellular ATP levels from 12 to 72 h (*n* = 3) are presented in **(A, B)**, respectively. The significance was calculated using Microsoft Excel (MS Office 365). *p < 0.05, **p < 0.01, and ***p < 0.001 indicate differences between treatment groups.

DAMPs are molecules that are secreted, discharged, or surface-exposed by perishing, stressed, or wounded cells. DAMPs might act as immune system adjuvants or warning signs of danger. The apoptotic stage and the kind of stress or cell death stimulus that triggers it determine the trafficking mechanism responsible for the production of ATP. Furthermore, it is possible that the methods and spatiotemporal pattern of ATP secretion from dying cancer cells play a crucial role in creating the proper extracellular ATP gradient, which is necessary to produce its chemotactic or DAMP-like activities. Extracellular ATP can promote cell growth and may contain immunosuppressive substances like adenosine. Pro-inflammatory ATP has the potential to hydrolyze into immunosuppressive adenosine, which could inhibit the immune system and create a milieu that supports tumor growth and decreases the effectiveness of the anti-tumor immune response. Figure 6 demonstrates that normal astrocyte cells’ ATP signals grow in response to HPM treatment. It was discovered that at 60 pulses, 24 h yield statistically meaningful results. In typical astrocytes, these events persist for up to 72 h (Figure 6A). While after receiving HPM therapy, U87-MG displays a distinct pattern. At 25 pulses beginning at 12 h, U87-MG exhibits reparable increases in ATP signals. After 24 h, there is a noticeable difference in the ATP Signals (Figure 6B).

### 3.5 Cell death

**FIGURE 7:**
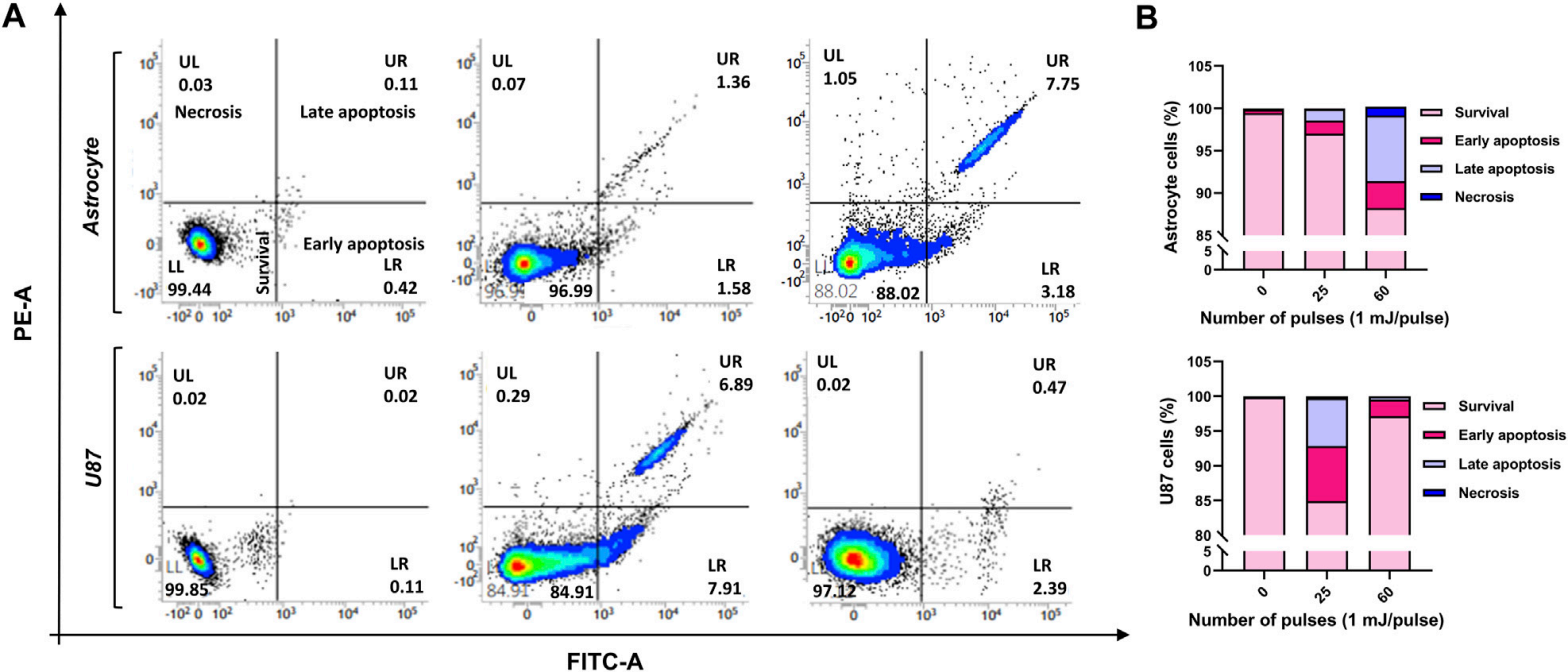
The cell death analysis. The effects of HPM exposure on the death of normal astrocytes in the brain and malignancies MG cells U-87 **(A)** FACS scatter plots of cell populations displaying propidium iodide fluorescence, suggesting uptake (*n* = 3). **(B)** Quantitative percentage of cell death after 25 and 60 pulses of HPM irradiation.

To examine if HPM exposure causes cell death in astrocytes and U-87 MG cells, the cells were seeded in 6-well plates, subjected to HPM radiation (25 and 60 pulses), and incubated for 24 h. The cell death induced by pulsed HPM exposure was assessed by PI uptake, which is suggestive of a damaged plasma membrane, and the findings are shown in Figures 7A, B. The results show that the HPM-exposed groups differ significantly from the control groups in both astrocytes and U-87 MG cell populations. In astrocytes, cell death was observed only at a high number of pulses (60 pulses) and remained unaffected at 25 pulses of HPM. Interestingly, significant cell death was observed specifically in 25 pulses of HPM in brain cancers U-87 MG. Such effects show an important sign which offers to investigate the mechanism involved for the cell death in dose-dependent manners in brain normal astrocytes and cancer U-87 MG cells. Following the HPM pulses 25 and 60, the overall rates of early and late apoptosis in astrocytes were 2.94% and 10.93%, respectively. Similar to this, after receiving HPM pulses 25 and 60, U-87 MG experienced a total early and late apoptosis percentage of 14.8% and 2.86%, respectively (Figure 7).

### 3.6 Intracellular ROS levels

**FIGURE 8:**
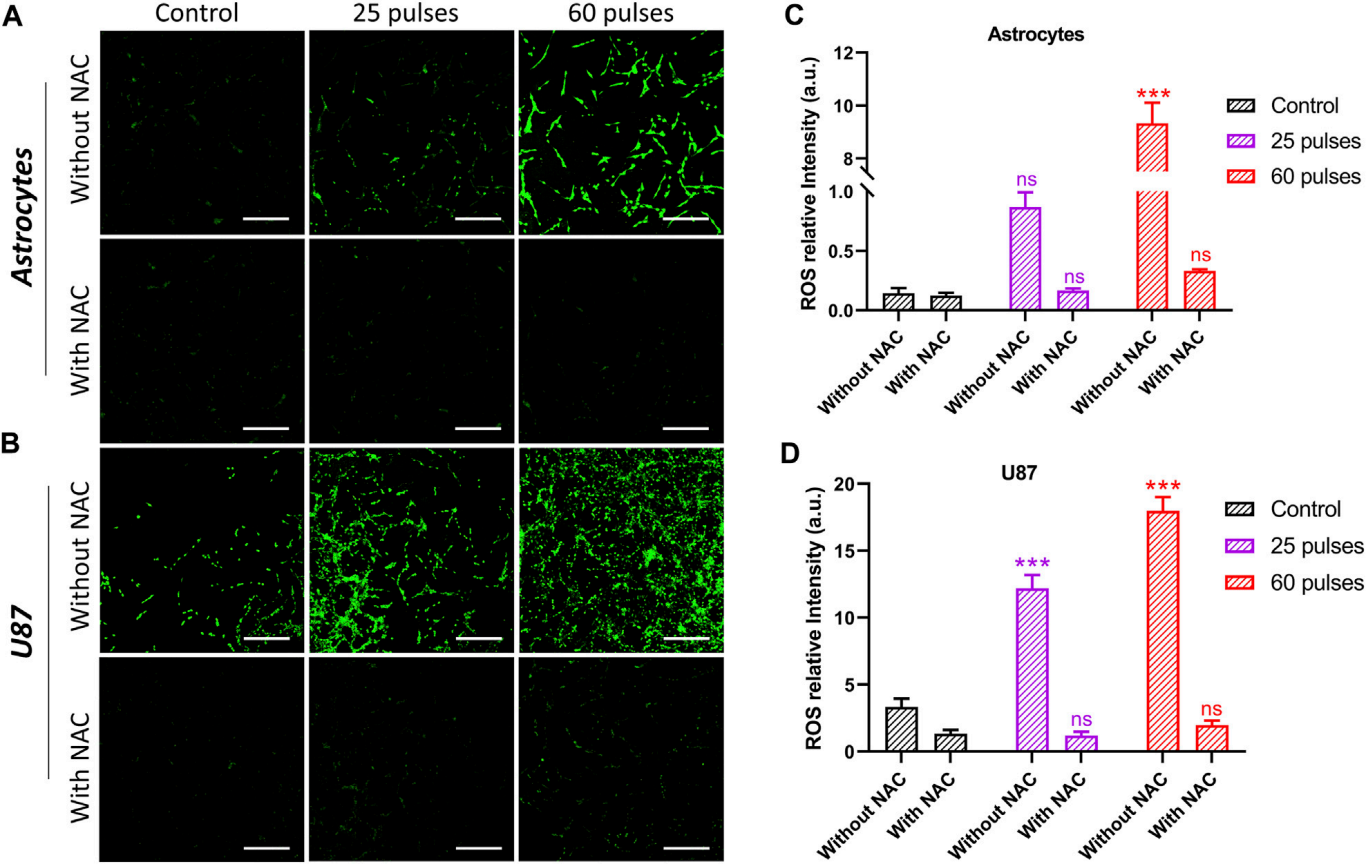
Intercellular ROS without and with NAC. **(A)** The intercellular ROS intensity in brain normal astrocytes without and with using NAC, and **(B)** indicates in brain cancer U87 cells, respectively. The **(C, D)** indicate the ROS relative intensity without and with NAC in astrocytes and U87 cell lines, respectively. Scale bar = 100 µm.

Figure 8 shows the intracellular ROS levels in control, treated with 25 pulses, and 60 pulses of HPM. The reduction of ROS levels was also measured by using intracellular ROS scavenger NAC. To determine the amount of NAC, we tested the cell viability of normal astrocyte and cancer U87 at various NAC concentrations, and the results are shown in Supplementary Figure S3. Based on the findings, we have chosen the NAC concentration of 5 mM for further investigation. Interactions with proteins in the mitochondrial porousness transition complex cause ROS to activate the intrinsic apoptotic cascade. ROS-induced oxidative stress causes mitochondrial depolarization. Pulsed HPM exposure can produce ROS, triggering apoptosis directly. Figures 8A, B shows that HPM irradiation resulted in considerably greater levels of ROS and RNS fluorescence and relative quantities of ROS and RNS levels in U87 MG cells than in untreated controls. The intracellular ROS in astrocytes was dramatically increased at 60 pulses, which is considered a very high dosage region and may be damaging to normal cells. The scavenger NAC reduced the ROS level in astrocytes to non-significant levels. Figure 8B shows that a comparable rise in intracellular levels was detected in U87 cell lines, which are normally higher than normal cells. The ROS level in U87 was reduced to non-significant after employing the scavenger NAC. Figures 8C, D show the intensity of intracellular ROS levels in brain normal astrocytes and cancer U87 cells without and with NAC scavenger.

### 3.7 Pulsed 3.5 GHz HPM-induced ROS production upregulates apoptotic markers

**FIGURE 9:**
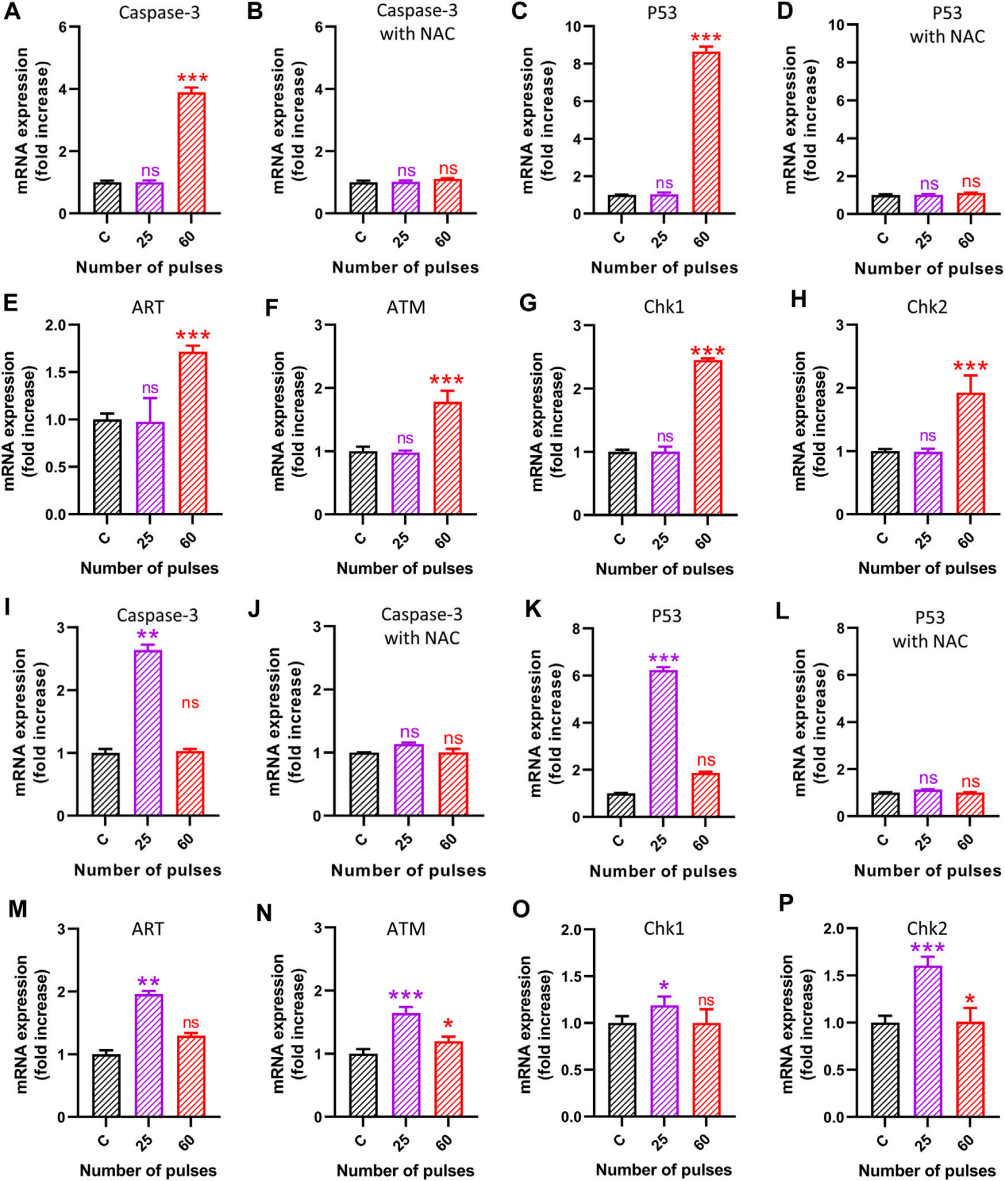
The molecular analysis 24 h after HPM exposure. The **(A–H)** indicates the gene analysis is astrocytes and **(I–P)** in brain cancer cells U87. **(A–B)** Caspase-3 without and with NAC in astrocytes, **(C–D)** p53 without and with NAC in astrocytes, **(E)** ART, **(F)** ATM, **(G)** Chk1, and **(H)** Chk2, in astrocytes **(I–J)** Caspase-3 without and with NAC in brain cancer U87, **(K–L)** p53 without and with NAC in brain cancer U87, **(M)** ART, **(N)** ATM, **(O)** Chk1, and **(P)** Chk2, in brain cancer U87.

The findings of the preceding studies indicate that HPM radiation had a considerable effect on astrocytes and U-87 MG cells at specific doses. We aimed to examine the mechanisms behind the various impacts on cells in this context. To that end, we performed quantitative PCR (qPCR) to investigate the effects of 3.5 GHz pulsed HPM on apoptosis-related molecular gene expression in astrocytes and U-87 MG cells. We experimented with different apoptosis-related molecular gene expressions to see if 3.5 GHz HPM irradiation may cause ROS generation in astrocytes and U-87 MG cells. The 3.5 GHz HPM irradiation increased ROS generation in astrocytes and U-87 MG at higher doses on cells and drastically altered gene levels of apoptotic markers 24 h after exposure. The HPM was utilized to deliver 25 and 60 pulses to astrocytes and U-87 MG. We observed that HPM irradiation significantly elevated apoptosis-related molecular markers when compared to the control group. For further confirmation to determine whether ROS has an impact on DNA damage, the p53 marker activation we treated cells with NAC (5 mM), a ROS scavenger, and saw decreased p53 activity after the treatment. The cell viability of normal astrocytes and cancer U-87 MG was also tested without and with NAC in control and HPM-treated groups, and the findings are shown in Supplementary Figure S4. The study of astrocytes’ apoptotic markers shown in Figure 9A–H shows that after 60 pulses, caspase 3, p53, ART, ATM, Chk1, and Chk2 levels considerably increased in astrocyte cells at higher doses. It is observed in Figures 9A–D, that the caspase-3 and p53 levels increased without NAC, and becomes non-significant when using NAC. Similar behavior was observed in U-87 MG cells but at a different dose (25 pulses) shown in Figures 9I–L. Additionally, as shown in Figures 9M–P, the levels of ART, ATM, Chk1, and Chk2 in brain cancer U87 MG cells significantly increased at 25 pulses in particular. In U87 MG cell line, the ATM and Chk2 are slightly upregulated at 60 pulses, but significance expression is only seen at 25 pulses. The results of our additional research into the expression levels of Caspase-8, Fas, MCP1, and PARP in both brain-normal astrocytes and brain-cancer U-87 MG cell lines are shown in Supplementary Figure S5. The Supplementary Figure S5 demonstrates that after HPM exposure, the expression of Caspase-8 and PARP appears to be increased (60 pulses in astrocytes and 25 pulses in U87). In both cell lines, HPM irradiation did not significantly affect Fas and MCP1 (Supplementary Figure S5). We verified p53 and caspase 3 activity following NAC treatment as well, allowing us to demonstrate that after scavenger its activity lowers directly affecting cell death events. In comparison to 60 pulses, 25 pulses in astrocytes did not significantly influence apoptotic markers. When determining whether ROS plays a significant role in DNA damage or not in the U87-MG cell line after scavenger treatment (NAC), we noticed that caspase 3 and p53 decreased noticeably when compared to non-treated groups. Accordingly, we demonstrate that ROS plays a significant role in inducing apoptosis inside cells. However, U87-MG exhibits different behavior in 60 pulses, during which all apoptotic markers decrease dramatically and effects become non-significant. The behavior of brain cancer cells towards HPM 40 and 60 pulses offers further exploration of the mechanism involved at higher doses in the future.

### 3.8 Pulsed 3.5 GHz HPM induced oxidative DNA damage

**FIGURE 10:**
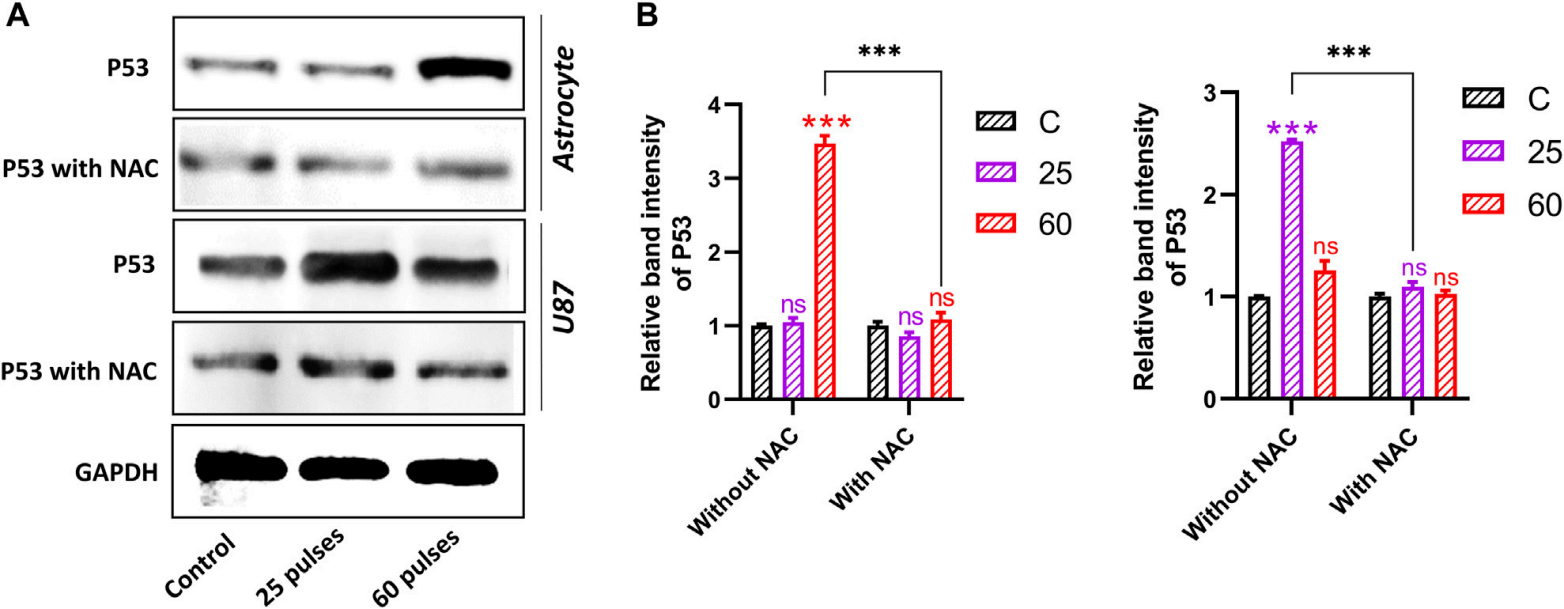
Western blot analysis without and with NAC. **(A)** The protein expression and activation of p53 in both brain normal astrocytes and cancer U87 cells 24 h after HPM exposure at specific doses. **(B)** The relative band intensity of astrocyte and U87 cells. The significance was calculated using Microsoft Excel (MS Office 365). *p < 0.05, **p < 0.01, and ***p < 0.001 indicate differences between treatment groups.

We observed the activation of p53 in response to the DNA damage and cause cell apoptosis owing to 3.5 GHz HPM exposure. Cells were collected for protein and mRNA extraction after the HPM irradiation to measure the degree of p53 alterations and the expression of genes that p53 targets. Western blotting research revealed that at 3.5 GHz, the level of p53 considerably rose. Our results demonstrated that 3.5 GHz HPM-induced increase in p53 activity. Figure 10A shows the normal astrocytes undergoing the p53-activated apoptotic pathway following higher doses of HPM which is considered a harmful dose. The band intensity is greater in 60 pulses than it is in 25 pulses. While p53 activation in U87-MG is stronger in 25 pulses compared to 60 pulses, the band intensity clearly shows p53 activation specifically in 25 pulses. The generation of ROS was reduced by the all-around antioxidant NAC. Figure 10B shows the band intensity without and with NAC, in control and HPM exposed groups. Astrocytes and U-87 MG cells were pre-treated with NAC and exposed to HPM to further support the idea that ROS formation is necessary for 3.5 GHz radio-induced DNA damage. After treatment with a 3.5 GHz HPM, we discovered that the DNA damage in astrocyte cells at 60 pulses and in U-87 MG cells decreased drastically (Figure 10). These findings suggest that astrocytes and U-87 MG cells exposed to 3.5 GHz HPM produce some ROS, which is in charge of DNA damage and nuclear condensation. Intriguingly, NAC dramatically reduced DNA damage in astrocytes and U87 MG cells.

**FIGURE 11:**
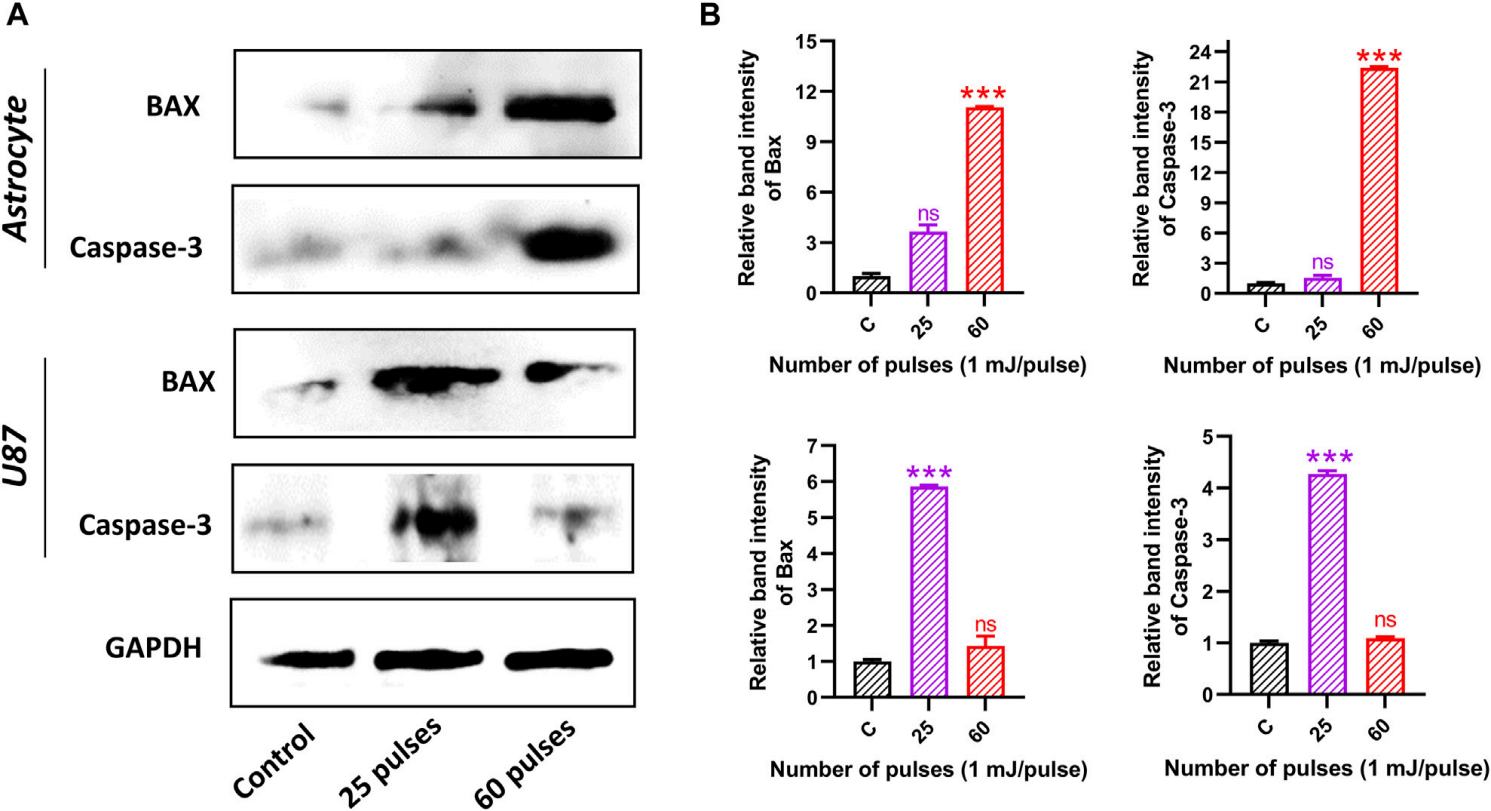
Western blot analysis. **(A)** The protein expression and activation of BAX and Caspase-3 in both brain normal astrocyte and cancer U87 cells 24 h after HPM exposure at specific doses. **(B)** The relative band intensity of astrocyte and U87 cells.

Furthermore, we have observed the protein expression levels of Bax and Caspase-3 in both brains’ normal astrocytes and brain cancer U87 cell lines 24 h after 25 and 60 pulses of HPM. In astrocytes, the band intensity of Bax and Caspase-3 was found to be increased at only higher doses (60 pulses). On the other hand, the band intensity in brain cancer U87 cells was found to be increased at 25 pulses as shown in Figures 11A, B indicates the relative band intensity (Bax and Caspase-3) of astrocyte and U87 in the control and treated groups. The obtained results from protein expression support our findings.

## 4 Discussions

To evaluate the biological effects of exposure to 3.5 GHz pulsed HPM radiation and the risks to human health, numerous types of research were conducted, producing a significant amount of data (Schwarz et al., 2008; Kesari et al., 2011). These statistics, however, appear to be at odds with one another, most likely as a result of the diverse biological systems’ sensitivity to radiation exposure and the various electromagnetic field parameters (Zhao et al., 2007; Schüz et al., 2011). Both aspects affect how microwave and living cells interact with one another (Mumtaz et al., 2022a). For instance, human breast epithelial MCF10A cells were not oxidatively stressed by single or repeated radiofrequency radiation exposure (Hong et al., 2012). The reproductive systems of both men and women may be considerably impacted by oxidative stress brought on by Wi-Fi or mobile phone EMR, according to recent studies (Mumtaz et al., 2022a). Additionally, EMR can influence gene expression and impact a variety of biological processes, including cell-cycle regulation, apoptosis, and autophagy (Mumtaz et al., 2022a). The current study shows that exposing astrocytes and U-87 MG cells to HPM at 3.5 GHz (power: 1 mJ/pulses) causes apoptosis in both cells but at different and specific doses.

Numerous cellular stressors, such as DNA damage, hypoxia, and nucleotide deficiency, can cause p53 to become activated. It has been demonstrated that p53 is activated by a variety of DNA damage types, including those caused by ionizing radiation (IR), radio-mimetic medicines, ultraviolet light (UV), and compounds like methyl methane sulfonate (MMS) (Lakin and Jackson, 1999; Hong et al., 2012; Carlsen and El-Deiry, 2021). Due to the exceedingly short half-life of the polypeptide, p53 levels are typically kept at a low level. Additionally, p53 generally exists in an essentially inactive state that is not very effective at binding to DNA and activating transcription. The ability of p53 to bind DNA and drive transcriptional activation is boosted, and its levels rise quickly, in response to DNA damage (Mukherjee et al., 2022).

**FIGURE 12:**
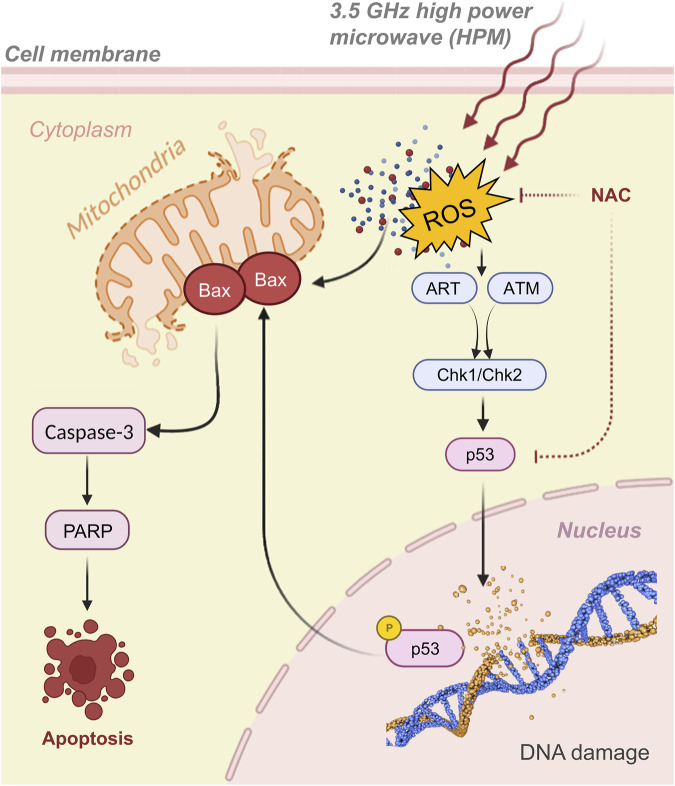
**T**he graphical representation of activation of p53, Bax, and caspase-3, as well as the signaling pathway, in which 3.5 GHz pulsed HPM, promote cell apoptosis. The 3.5 GHz pulsed HPM activates the p53 signaling pathway through the ART/ATM pathways which leads to the activation of Chk1/Chk2 by increasing ROS production and DNA damage. p53 then upregulates Bax and caspase cascades, resulting in cell apoptosis.

Following that, several genes that produce apoptosis, DNA repair, or cell-cycle arrest are activated (Lakin and Jackson, 1999). In our investigation, we found that activation of the mitochondrial Bax and caspase cascades causes DNA damage in response to HPM, which then may cause apoptosis *via* an intrinsic mechanism. One such scenario is that HPM-induced ROS production activates the intrinsic pathway and initiates apoptosis and possible mechanism pathways as shown in Figure 12. The activation of Bax, Cytochrome c, ATM, and the decrease in Bcl-2, which is further linked to the activation of p-53, cause DNA damage and apoptotic cell death, which has been well established from previous studies (Sun et al., 2005; Chen et al., 2010; Fan et al., 2013; Jiang et al., 2021; Kudo et al., 2022). In this study, we found that p53 expression increased and that Bax and caspase-3 were both activated. According to the currently available literature and our findings, it is reasonable to assume that the ART/ATM pathway, which is supported by molecular analysis Figure 9, is responsible for the rise in p53 expression following HPM exposure. Due to their role in numerous clinical disorders, mitochondria-derived ROS have attracted growing attention. In our investigations, we observed variable ROS generation in astrocytes and U-87 MG cells (Figures 8A–D). This behavior differs in normal cells, where at 60 pulses it damages the cells, although in U-87 -MG, where at 25 pulses viability is severely decreased. These findings suggest that at substantial pulses, this HPM exhibits different behavior in cancers. We hypothesize that ROS formation and DNA damage may have a synergistic effect in controlling 3.5 GHz HPM-induced p53 activation and cell death. Since 3.5 GHz HPM can also directly induce DNA damage, which will further boost p53 activation. We found certain damage-associated molecular pattern (DAMPs) indicators even after 3.5 GHz treatment (Figures 6A, B), which are secreted by cells under particular instances. The most frequent marker for DAMPs analysis is ATP. As shown in Figures 5, 6, we measured the intracellular and extracellular ATP released to confirm that, following 3.5 GHz HPM treatment, the cells experienced external stress at various pulse rates in normal astrocytes and cancer U87 cells. Interestingly, in U87-MG, 25 pulses are specific to cause a reaction that releases ATP outside the cell because of cell destruction. Gamma knife radiosurgery (GKRS) is a unique approach that is being utilized and used in patients to treat cancers/tumors (Samanci et al., 2022; Shin et al., 2022). Pituitary adenomas can be treated with GKRS although the procedure can result in significant hormone deficiencies (Palmisciano et al., 2022). The findings of this research might be beneficial in radiation to have an inhibitory impact on tumors at a specified dosage. Further research on this area is desperately required utilizing this particular frequency. Following 3.5 GHz HPM treatment at the different numbers of pulses (25 pulses in U87 and 60 pulses in astrocyte), demonstrate cell damage.

Since microwave exposure can also directly cause DNA damage, which will help enhance p53 activation (Shahin et al., 2015; Mukherjee et al., 2022). We hypothesize that ROS generation and DNA damage may work in concert to control 3.5 GHz HPM-induced p53 activation and cell death. More extensive studies and results are required to make a statement whether ROS are mainly responsible for to cause of DNA damage while exposed to pulsed HPM radiations. It is also unclear how ROS might influence apoptosis. Here, we found that at various pulse rates, 3.5 GHz HPM irradiation produces ROS production, which breaks DNA in astrocytes and U-87 MG cells and results in cell death (Figure 7). Furthermore, since NAC did not completely prevent the DNA damage caused by our experiment’s 3.5 GHz HPM irradiation, it is possible that this frequency directly causes DNA damage. ROS may have played a part in our work; therefore, we used an NAC scavenger to monitor their activity. Additionally, we can see intracellular ROS measurements inside cells in Figure 8 following 3.5 GHz HPM treatment at specific pulses, which may have caused apoptosis. We employed a scavenger to confirm its activity, and after NAC treatment a significant decrease in the amount of ROS inside the cells was found (Figure 8).

Although the p53 protein has been connected to a number of physiological functions, its function in microwave-induced cell death is still unclear. Here, we discovered that p53 was suppressed by using NAC and p53 target gene generated by 3.5 GHz HPM irradiation, indicating that ROS is involved in cell death (Figure 9). HPM-induced cell death is represented molecularly by the activation of the caspase-3 signaling cascade *via* p53. By increasing ROS production and DNA damage, 3.5 GHz HPM stimulates the p53 signaling pathway. P53 then upregulates BAX-mediated caspase-3 activation, which causes cell apoptosis as shown in Figure 12. HPM Induces the expression of p53/Caspase-3 dependent apoptosis (Figure 9). Numerous other cues besides ROS can also activate p53 (Mukherjee et al., 2022). Since 3.5 GHz HPM irradiation-induced p53 activation was partially inhibited by NAC in the current study, it is reasonable to hypothesize that ROS burst and DNA damage together may be responsible for upregulating p53 expression during 3.5 GHz HPM irradiation-induced apoptosis in astrocytes at the harmful dosage and may promoting DNA repair after 25 pulses in U87—MG directly by upregulating the expression of DNA repair genes which needs further exploration in future.

The Bcl-2 family of proteins controls apoptosis through the mitochondrial permeability transition, which is recognized to be a key feature in the antitumor response to cancer therapy (MPT). Mitochondrial membrane permeabilization (MMP)-inducing anion channel is inserted by the activated Bax/Bak in response to DNA damage (Lakin and Jackson, 1999; Yamaguchi et al., 2004; Hong et al., 2012). There is some evidence that suggests Bax, a proapoptotic gene belonging to the cell death-regulating bcl-2 family, may be implicated in p53-induced apoptosis. It has been demonstrated that p53 controls the transcription of the Bax gene, which has p53 consensus sequences within its promoter. In this investigation, we found that the HPM-induced formation of ROS had impacts on the mitochondrial membrane, including BAX. Our results demonstrated that 3.5 GHz HPM-induced increase in caspase-3 activity. Caspase-3 activation and cytochrome c release from the mitochondrion are essential events in cell death. We can observe that at 60 pulses, Bax and caspase-3 activations are higher in normal astrocytes which are considered harmful doses (higher dose). We can see that its activity is minimal from band intensity in 25 pulses. While in U87, at 25 pulses, the behavior of the MG changes. At 25 pulses, Bax and caspase 3 create a signal that leads to apoptosis. However, it exhibits a different behavior while lowering its band intensity at 60 pulses.

Redox-controlling genes may be activated by p53-mediated apoptosis, producing ROS. In this investigation, we found evidence to support the idea that oxidative stress brought on by 3.5 GHz HPM irradiation is a key factor in controlling p53-dependent caspase-3 activation. Cysteine proteases called caspase-3 have a well-known role in inducing apoptosis. It is well recognized that proapoptotic substances like cytochrome c produced from mitochondria can activate caspase-3. ROS are primarily produced by mitochondria at the level of respiratory chain complexes I and III. Our findings from the current study indicated that 3.5 GHz HPM caused the release of Bax and caspase-3 activation (Figure 11). The observation that NAC was able to prevent p53 from acting (Figure 10) demonstrates how ROS activation causes p53 action to be induced within the cell and may also signal the activation of the mitochondrial Bax. Therefore, it is safe to say that 3.5 GHz HPM irradiation may cause mitochondrial ROS and the release of Bax, which in turn activates caspase-3 in astrocytes and U-87 MG cells at specific doses.

## 5 Conclusion

Humans are swimming like fish in a large ocean of EM radiation, exposing them on a regular basis. The biological effects and safety level of pulsed HPM are becoming a serious issue as the number of its applications expands with time. Fewer studies on pulsed HPM have been conducted, and the molecular processes behind the influence of HPM on the brain remain largely unclear. The purpose of this study was to look into the impact of 3.5 GHz pulsed HPM radiation on the survival of human brain cancer and normal cells. Cell survival, mitochondrial activity, intracellular ROS production, DNA damage, ART/ATM, Chk1/Chk2, p53 expression (without and with NAC), Bax, and caspase-3 activity were all assessed after HPM treatment. We found that 3.5 GHz HPM exposure produces ROS formation within the medium and inside the cell due to the intense electric field of ∼23 kV/cm, as well as DNA damage in astrocytes and U-87 MG cells. The brain normal astrocyte was found to be unaffected until 40 pulses which might be the threshold level of HPM exposure. Exceeding this threshold of exposure lead to harmful effects on the brain. Furthermore, we discovered the dosage range at which U87-MG inhibits while leaving normal cells unaffected, which is important from a therapeutic aspect as well. Interestingly, a significant decline in the viability of brain cancer cells was achieved specifically at 25 pulses which lies within the safe level for normal cells. These findings are important for understanding the physiological mechanisms underlying HPM-induced cell death, as well as the acceptable range for HPM exposure on normal cells and therapeutic effects on cancer U87. As pulsed HPM technology progresses, we believe this study is timely to aid humanity and future research.

## Data Availability

The original contributions presented in the study are included in the article/Supplementary Material, further inquiries can be directed to the corresponding authors.
