# Supplementary material for: ROS production in response to high-power microwave pulses induces p53 activation and DNA damage in brain cells: Radiosensitivity and biological dosimetry evaluation
Source: Front Cell Dev Biol. 2023 Feb 23;11:1067861. doi: 10.3389/fcell.2023.1067861 (PMC9996137; doi:10.3389/fcell.2023.1067861)
Supplement: Supplementary file 1 [file DataSheet1.PDF]

Supplementary Material

ROS production in response to high-power microwave pulses induces p53 activation and DNA damage in brain cells: Radiosensitivity and biological dosimetry evaluation

1 Formation of virtual cathode and phase space of electron inside HPM generator device

A dense virtual cathode (VC) was formed when the injected beam current I_{beam} sufficiently exceeds the critical value of space-charge limiting current I_{SCL}. The approximate value of I_{SCL} using a solid beam of radius r_o in a waveguide having an inner radius R can be calculated by using a famous formula expressed as (Bogdankevich and Rukhadze, 1971):

$$I_{SCL} = I_A \frac{(\gamma^{\frac{2}{3}} - 1)^{3/2}}{1 + 2\ln\left(\frac{R}{r_o}\right)} [kA] \quad (1)$$

where γ shows the beam relativistic factor and $I_A = 17\ kA$ is the Alfven current; thus, an essential condition $I_{beam} \gg I_{SCL}$ needs to be satisfied for the formation of a VC (Jiang et al., 1995; Choi et al., 2000). To verify the formation of VC, the phase space of electrons was monitored in simulation, and the results were shown in Figure S1 (A – D). The VC was successfully formed where the electrons are reflected at the anode position as shown in Figure S1 (D).

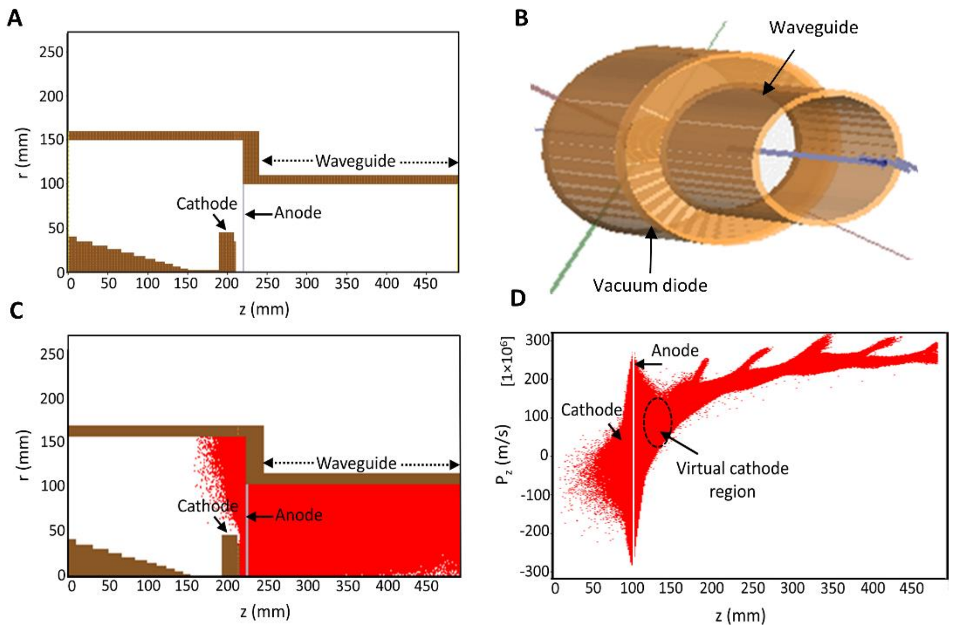

Supplementary Figure S1. The results were obtained from the 3D PIC simulation. The vircator 2D view and 3D view which are designed in PIC simulation were shown in (A) and (B), respectively.

The emission of the electron beam from the cathode surface was shown in (C), and (D) shows the (radial and axial) phase space of all electrons and the formation of a virtual cathode inside the device.

2 The emission mode of the HPM from three-dimensional particle-in-cell (3D PIC) simulation

The mode of HPM is crucial to understanding its nature, propagation, and its impact on biological systems. In PIC simulation from MAGIC, we found the HPM emission mode from the vector electric and magnetic field profiles as shown in Figure S2. The vector field profiles indicates that the TM_{01} is dominant in this study. The vector field profiles show that the TM_{01} is the dominant mode in this study. Axial vircators exhibit dominant TM mode due to axial vibrations of electrons and VC inside the drift tube region. The results obtained to analyze the mode of HPM are consistent with previous simulation and experimental results (Choi et al., 2000; Mumtaz, Sohail; Uhm, Hansup; Lim, Jun Sup; Choi, 2022).

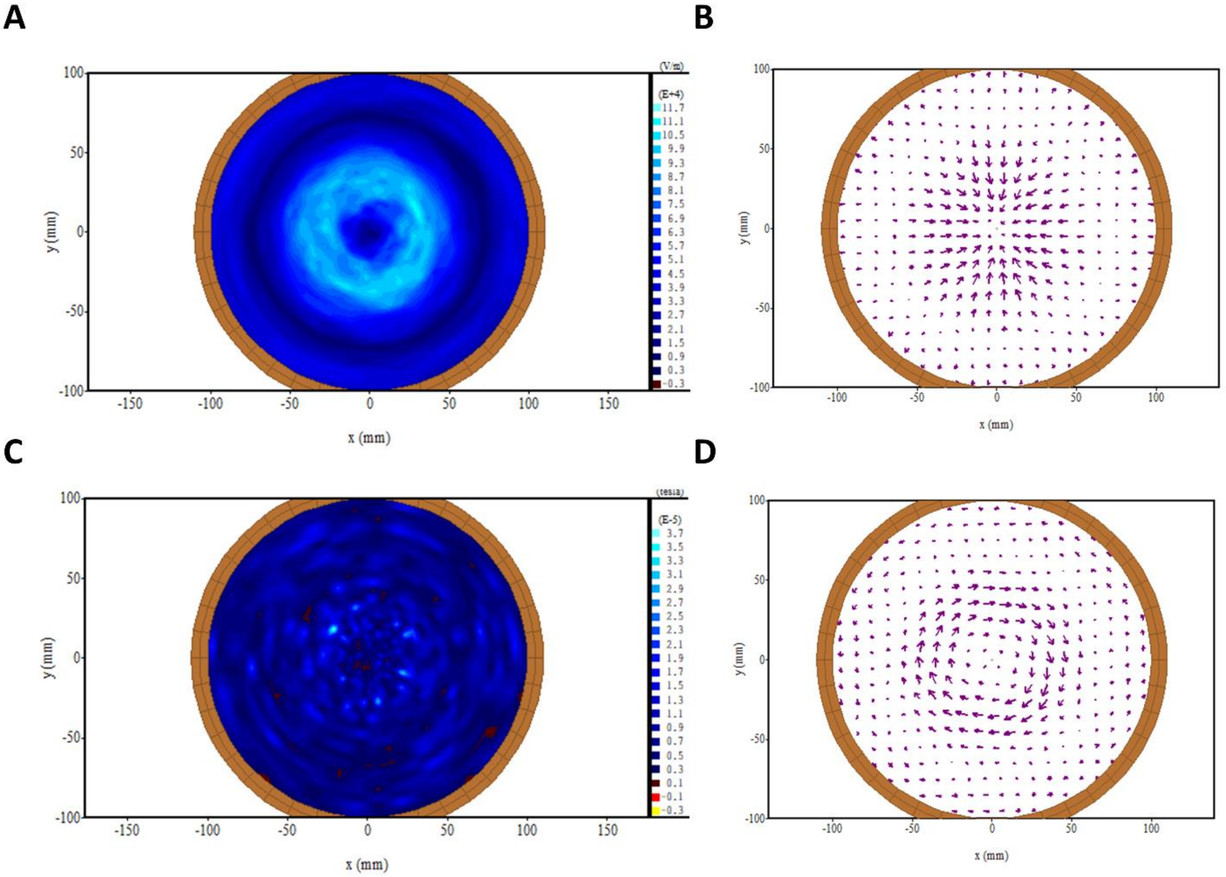

Supplementary Figure S2. The HPM electric and magnetic field were observed at the end of the waveguide at 35 ns simulation time. (A) Electric field distribution inside the waveguide region when HPM is already generated. (B) Vector electric field profile. (C) Magnetic field distribution inside the drift tube region at 35 ns simulation time. (D) Vector magnetic field. In vector electric and magnetic fields, the arrow strength shows the field strength and the direction of the arrow is the field direction. From the vector field profiles, the mode of the HPM was estimated as the dominant TM_{01}.

3 The cell viability by taking the different concentrations of NAC (ROS scavenger)

A

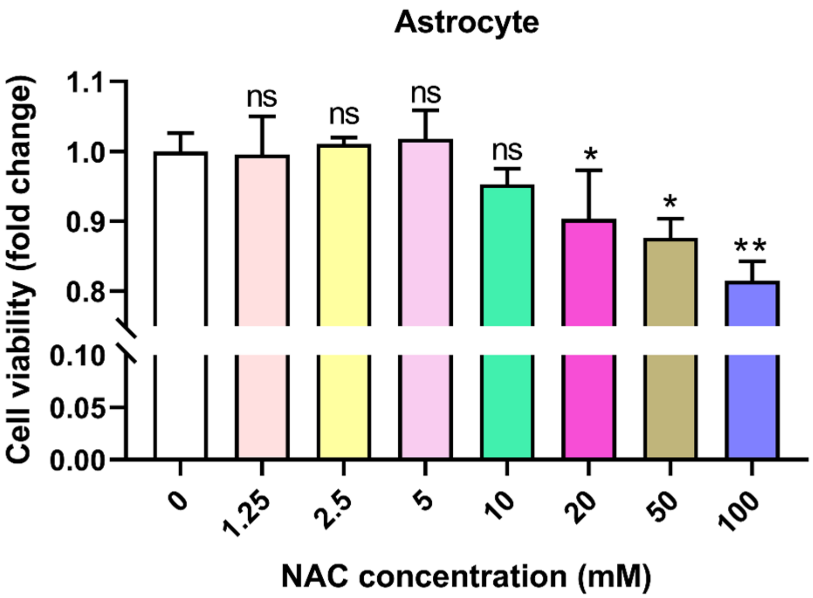

B

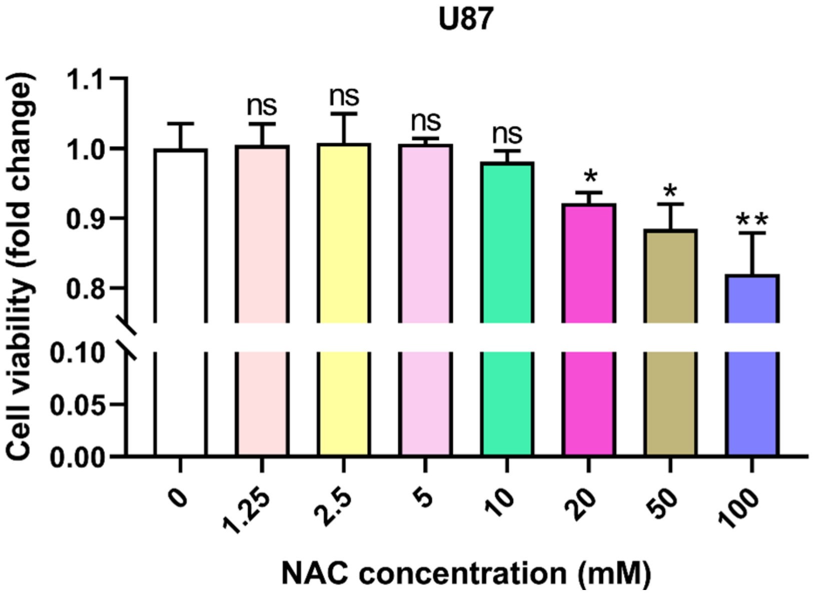

Supplementary Figure S3. The cell viability with different concentrations of scavenger NAC. (A) Astrocyte and (B) U87. The viability of the both cell lines remained unchanged up to 10 mM concentration of NAC. From these results, 5 mM was selected for the further analysis.

4 The cell viability without and with NAC before and after HPM irradiation

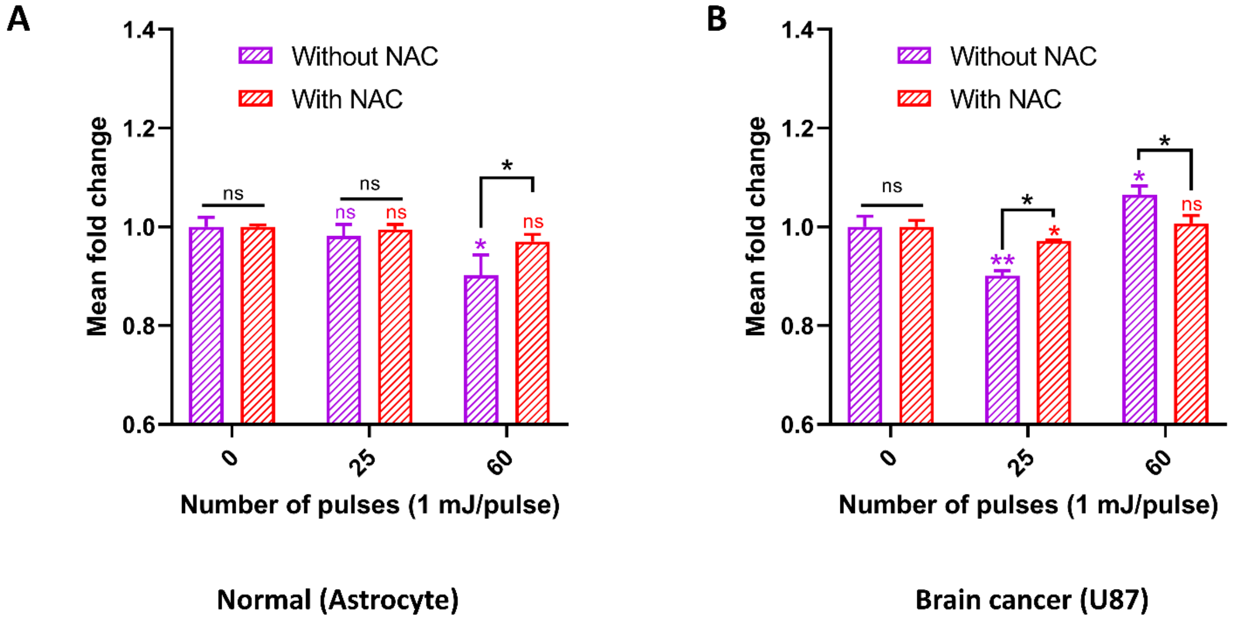

Supplementary Figure S4. The cell viability in control and (25 and 60 pulses of HPM) treated groups without and with NAC (ROS scavenger). (A) The viability of normal astrocyte cells in the brain, and (B) the viability of U87 cancer cells in the brain. The results show that the decreased viabilities were regained when the ROS scavenger NAC was used. These data imply that ROS played an important role in the loss of cell viability and apoptosis following HPM exposure.

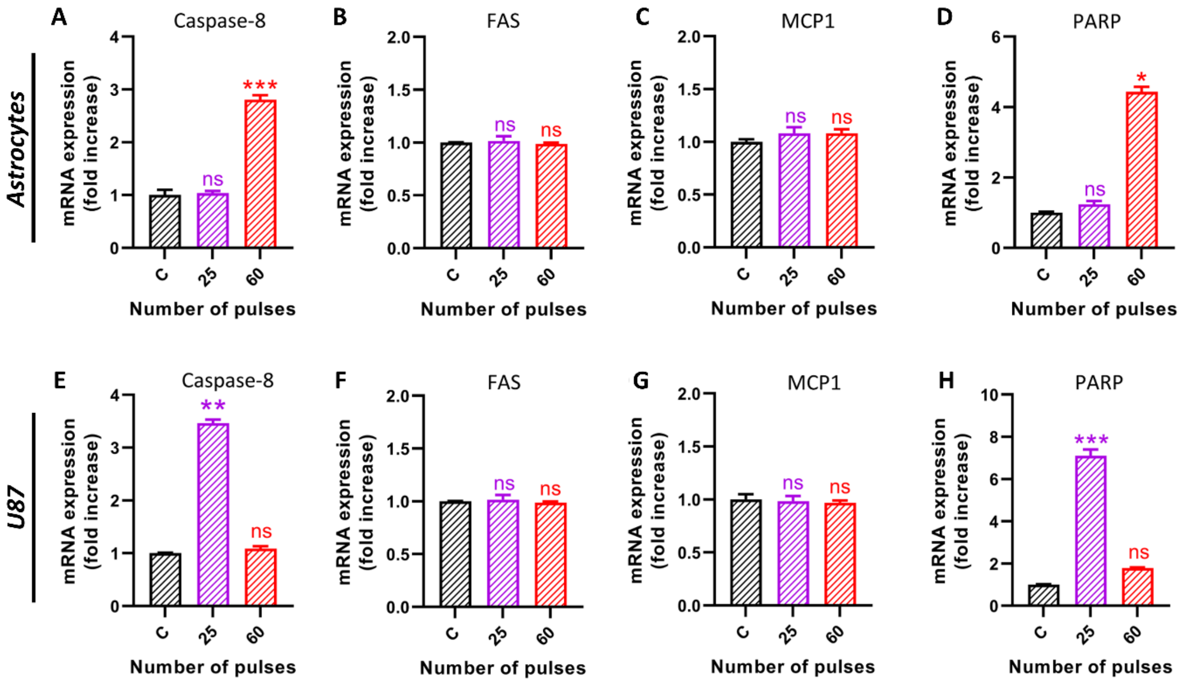

Figure S5. The molecular analysis 24 h after HPM exposure. The (A – D) indicates the gene analysis is astrocytes and (E – H) in brain cancer cells U-87 MG. (A) caspase-8, (B) FAS, (C) MCP1, and (D) PARP, in astrocytes. (E) caspase-8, (F) FAS, (G) MCP1, and (H) PARP, in brain cancer U-87 MG. It has been shown that HPM exposure increases the expression of Caspase-8 and PARP (60 pulses in astrocytes and 25 pulses in U-87 MG). FAS and MCP1 were unaffected by HPM irradiation in both cell lines at selected doses.

References

Bogdankevich, L. S., and Rukhadze, A. A. (1971). Stability of relativistic electron beams in a plasma and probelm of critical currents. *Sov. Phys. Uspekhi* 14, 163. doi: https://doi.org/10.1070/PU1971v014n02ABEH004456.

Choi, E.-H., Choi, M.-C., Jung, Y., Choug, M.-W., Ko, J.-J., Seo, Y., et al. (2000). High-power microwave generation from an axially extracted virtual cathode oscillator. *IEEE Trans. Plasma Sci.* 28, 2128–2134. doi: 10.1109/27.902240.

Jiang, W., Masugata, K., and Yatsui, K. (1995). Mechanism of microwave generation by virtual cathode oscillation. *Phys. Plasmas* 2, 982–986. doi: 10.1063/1.871377.

Mumtaz, Sohail; Uhm, Hansup; Lim, Jun Sup; Choi, E. H. (2022). Output-power enhancement of vircator based on second virtual cathode formed by wall charge on a dielectric reflector. *IEEE Trans. Electron Devices*. doi: 10.1109/TED.2022.3149455.
